# Evolution at two time frames: Polymorphisms from an ancient singular divergence event fuel contemporary parallel evolution

**DOI:** 10.1371/journal.pgen.1007796

**Published:** 2018-11-13

**Authors:** Steven M. Van Belleghem, Carl Vangestel, Katrien De Wolf, Zoë De Corte, Markus Möst, Pasi Rastas, Luc De Meester, Frederik Hendrickx

**Affiliations:** 1 Directorate Taxonomy and Phylogeny, Royal Belgian Institute of Natural Sciences, Brussels, Belgium; 2 Terrestrial Ecology Unit, Biology Department, Ghent University, Ghent, Belgium; 3 Department of Biology, University of Puerto Rico, Rio Piedras, Puerto Rico; 4 Department of Zoology, University of Cambridge, Cambridge, United Kingdom; 5 Institute of Ecology, University of Innsbruck, Innsbruck, Austria; 6 Ecological Genetics Research Unit, Department of Biosciences, University of Helsinki, Helsinki, Finland; 7 Laboratory of Aquatic Ecology, Evolution and Conservation, KU Leuven, Leuven, Belgium; Aarhus University, DENMARK

## Abstract

When environments change, populations may adapt surprisingly fast, repeatedly and even at microgeographic scales. There is increasing evidence that such cases of rapid parallel evolution are fueled by standing genetic variation, but the source of this genetic variation remains poorly understood. In the saltmarsh beetle *Pogonus chalceus*, short-winged ‘tidal’ and long-winged ‘seasonal’ ecotypes have diverged in response to contrasting hydrological regimes and can be repeatedly found along the Atlantic European coast. By analyzing genomic variation across the beetles’ distribution, we reveal that alleles selected in the tidal ecotype are spread across the genome and evolved during a singular and, likely, geographically isolated divergence event, within the last 190 Kya. Due to subsequent admixture, the ancient and differentially selected alleles are currently polymorphic in most populations across its range, which could potentially allow for the fast evolution of one ecotype from a small number of random individuals, as low as 5 to 15, from a population of the other ecotype. Our results suggest that cases of fast parallel ecological divergence can be the result of evolution at two different time frames: divergence in the past, followed by repeated selection on the same divergently evolved alleles after admixture. These findings highlight the importance of an ancient and, likely, allopatric divergence event for driving the rate and direction of contemporary fast evolution under gene flow. This mechanism is potentially driven by periods of geographic isolation imposed by large-scale environmental changes such as glacial cycles.

## Author summary

Evidence has accumulated that populations may adapt surprisingly fast to changing environments. This rapid, and often parallel, ecological adaptation is presumed to be facilitated when selection acts on preexisting genetic variation. However, the origin of this variation remains to be identified. In our work on genome-wide parallel divergence in a mosaic of two beetle ecotypes, we identify the genomic regions involved in adaptation. We show that the origin of the divergent alleles at different loci can be traced back to a singular, ancient divergence event. This event likely occurred in geographic isolation after which admixture of the diverged populations resulted in a polymorphic population that survived during the most recent glacial maxima. The alleles involved in adaptation to the alternative environments are currently present in much higher frequencies in the populations than generally assumed. Therefore, when habitats become available, the presence of these alleles may result in rapid and parallel ecological differentiation by the reassembly of these ancient alleles. We suggest that this mechanism may be common to examples of parallel evolution and might reconcile different views on the role of geographical isolation in ecological divergence.

## Introduction

Adaptation to local environmental conditions may lead to the evolution of distinct ecotypes and, ultimately, new species [1,2]. Under prolonged periods of geographical isolation, the absence of gene flow allows populations to accumulate new alleles by mutation and build-up genome-wide differences in the frequency of these alleles [3,4]. However, increasing evidence demonstrates that ecological divergence may occur surprisingly fast and even in absence of a physical barrier [5–8]. As new beneficial mutations are unlikely to accumulate rapidly, these cases of fast adaptation likely involve selection on standing genetic variation, i.e. genetic variation that was present in the ancestral population before divergence took place [9–11]. Characterizing the origin and factors that maintain standing genetic variation is important as it can help understand the rate and direction of genetic adaptation to rapid environmental change [10,12,13].

**Fig 1:**
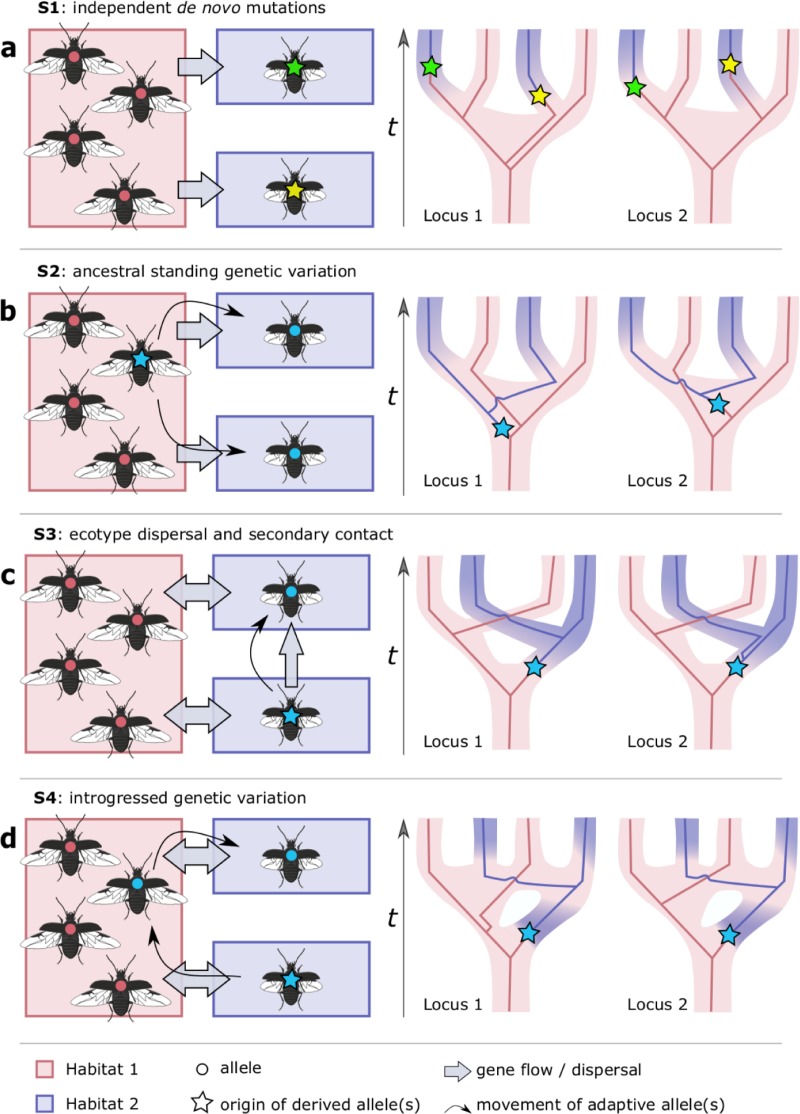
Evolutionary scenarios describing the origin of adaptive alleles in cases of parallel ecotypic divergence. **S**chematics on the left show the colonization of a new ‘blue’ habitat and the origin of alleles adapted to this new habitat. On the right, population histories are shown with examples of the expected genealogies at two unlinked loci that include an adaptive allele. **(a.)** In scenario **S1**, repeated adaptation to the ‘blue’ habitat occurs through independent *de novo* mutations and genealogies will not show monophyletic clustering of alleles adapted to the ‘blue’ habitat. **(b.)** In scenario **S2**, mutations originate as rare neutral or mildly deleterious alleles within the ancestral population and are later repeatedly selected when populations are exposed to the alternative environmental condition. This will be evident by monophyletic clustering of alleles adapted to the ‘blue’ habitat, but divergence patterns at unlinked loci that include an adaptive allele may differ strongly. **(c.)** In scenario **S3**, the derived ecotype evolves in geographic isolation, but disperses into suitable habitat patches and comes repeatedly into secondary contact with the ancestral ecotype. **(d.)** In scenario **S4**, derived alleles initially arise within a single isolated population as in S3, but are introgressed into the ancestral population, providing the raw genetic material for repeated and rapid evolution when populations later face similar environmental conditions. For both S3 and S4 monophyletic clustering of alleles adapted to the ‘blue’ habitat is expected, as well as a shared divergence pattern across unlinked selected loci.

Populations that have recently and repeatedly adapted to similar ecological conditions (i.e. parallel adaptation) hold the promise to identify the loci and alleles involved in ecological divergence [14–16]. However, the origin of the alleles that allow populations to repeatedly adapt to the alternative environment generally remains poorly characterized and different evolutionary scenarios can be proposed [17]. A first scenario comprises repeated adaptation through independent *de novo* mutations that occur within the alternative environment (Figs 1A and S1). Alternatively, several scenarios describe repeated adaptation from standing genetic variation (Figs 1B–1D and S2–S4). In a second scenario, mutations originate as rare neutral or mildly deleterious alleles within the ancestral population and are repeatedly selected when populations become exposed to the alternative environmental condition (Figs 1B and S2) [18]. In a third scenario, the derived alleles initially evolve within a single isolated population that is exposed to the alternative environment and later disperse to come repeatedly into secondary contact with the ancestral ecotype (Figs 1C and S3). Similarly, in a fourth scenario, the derived alleles evolve in isolation, but secondary contact and admixture with the ancestral population may then result in polymorphism at these adaptive loci. These polymorphisms can then provide the raw genetic material for repeated and rapid evolution when populations later face similar environmental conditions (Figs 1D and S4) [19–21]. This latter scenario is distinct in that rapid and repeated ecological divergence results from evolution at two different time frames, in the sense that contemporary adaptation is based on alleles that evolved during an ancient divergence in geographic isolation.

It should be possible to discriminate amongst the alternative scenarios that describe the origin of standing genetic variation by integrating patterns of pairwise differentiation with properties of the gene genealogies at multiple unlinked loci as well as models that describe the demographic history of the populations [22]. If alleles involved in adaptation evolved through independent mutations, they are expected to occur at different loci or at random along the genealogy within a single locus (Figs 1A and S1). Therefore, they will not be identical-by-descent, because adaptive *de novo* mutations can occur on different haplotypes in different geographic regions. Alternatively, if ecological differentiation is based on alleles that are present as standing genetic variation in the ancestral population, the derived alleles are expected to be identical-by-descent, but their evolutionary history may differ strongly at unlinked selected loci (Figs 1B and S2). Next, if adaptive alleles evolved initially within an isolated population and later came into repeated secondary contact with the ancestral ecotype, the initial evolution of the entire ecotype has a singular evolutionary origin and a shared divergence pattern is expected across unlinked selected loci (Figs 1C and S3). Gene-flow at secondary contact may in this scenario swamp the initial neutral genetic differences and only genomic regions involved in adaptive divergence are expected to withstand the homogenizing effect of gene flow. However, a highly similar genomic pattern could emerge if the derived ecotype evolved in geographic isolation and adaptive alleles were later reintroduced into the source population (Figs 1D and S4) [21,23–25]. Therefore, distinguishing scenario S3 from S4 requires additional lines of evidence that demonstrate repeated secondary contact with only gene flow at neutral loci rather than introgression of derived alleles in the ancestral population and subsequent more recent *in situ* genetic divergence from these introgressed alleles.

**Fig 2:**
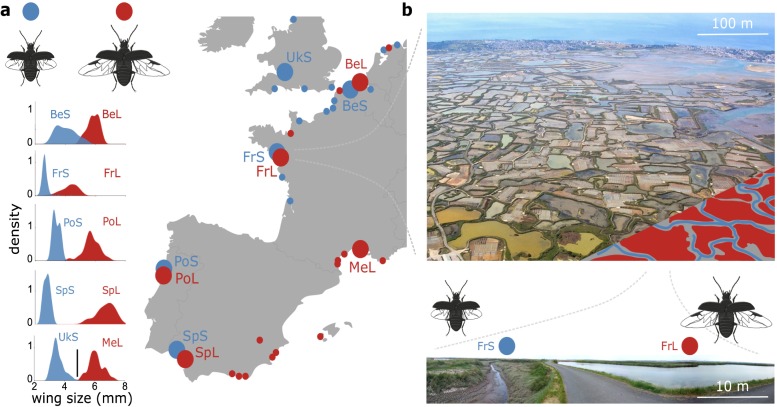
*Pogonus chalceus* sampling and ecotypic divergence. **(a.)** Sampling locations and density plots of the wing size distribution in the sampled populations. Blue indicates tidal habitats with short-winged beetles, red indicates seasonal habitats with long-winged beetles. BeS: Belgium-short, BeL: Belgium-long, FrS: France-short, FrL: France-long, UkS: UK-short, MeL: Mediterranean-long, PoS: Portugal-short, PoL: Portugal-long, Sps: Spain-short, SpL: Spain-long. Large circles represent populations included in the present study, small circles represent populations sampled previously [28,36]. **(b.)** Detail of the Fr population (Guérande) being a historic salt-extraction area that was created by man approximately one thousand years ago and consists of a network of tidal inundated canals (indicated in blue in the lower right corner) interlaced with seasonally inundated salt extraction ponds (partly indicated in red in the lower right corner) (image courtesy by Alexandre Braun). Populations of the short- (FrS) and long-winged (FrL) ecotype are found in the tidal (blue) and seasonally (red) inundated habitats, respectively, and occur in close proximity (< 20 m) within this sympatric mosaic. The bottom image shows a panoramic detail demonstrating the close proximity of the tidal (left) and seasonally flooded (right) sampling locations.

Populations of the saltmarsh beetle *Pogonus chalceus* provide an interesting case to study parallel evolution [26]. *Pogonus chalceus* beetles have adapted to two contrasting habitat types across Atlantic-Europe; tidal and seasonal salt marshes (Fig 2A). *Tidal* salt-marshes are inundated on an almost daily basis for at most a few hours and are inhabited by *P*. *chalceus* individuals that have a relatively small body size, short wings and submergence behavior during inundation. In contrast, salt-marshes that are subject to *seasonal* inundations that last for several months, harbor *P*. *chalceus* individuals with a larger body size, fully developed wings and more frequent dispersal behavior upon inundation [27–29]. Although these ecotypes diverged in multiple traits towards these contrasting hydrological regimes, we mainly refer to them as the short-winged tidal and long-winged seasonal ecotype, respectively, in accordance with previous studies [27–29]. Populations of both ecotypes can be found along the Atlantic coastal region in Europe and often occur in close proximity and even sympatric mosaics (Fig 2B) [30]. In the sympatric mosaics, contrasting behavioral adaptations towards the inundation regimes in the tidal and seasonal marshes potentially result in different habitat preference of the ecotypes and may present an incipient reproductive isolating mechanism [29]. Despite evidence that divergence in wing size in this species is polygenic and under strong genetic control [28,30,31], previous research based on microsatellite data also revealed very low neutral genetic differentiation between the ecotypes within geographic locations [30]. This suggests either a very recent differentiation and/or high levels of ongoing gene flow between these ecotypes. At least for wing-size, a fast rate of *in situ* evolution is corroborated by the observation of a clear reduction in wing size in a small isolated tidal marsh that has been colonized by long-winged individuals less than two decades ago (S1 Supporting Results).

To infer the origin of the allelic variants that underlie parallel evolution in *P*. *chalceus*, we here investigate genomic differentiation in multiple ecologically divergent population pairs and reconstruct the evolutionary history of the alleles underlying ecological divergence. In agreement with an ancient singular divergence event, we find sharing of the genealogical pattern at unlinked loci that show signatures of selection. Moreover, the apparent potential of these beetles to rapidly and repeatedly adapt to the different tidal and seasonal hydrological regimes, is likely fueled by the maintenance of relatively high frequencies of alleles selected in the alternative habitat. These results contribute to our understanding of the mechanisms underlying fast and parallel ecological adaptation and the factors determining the evolutionary potential of populations and species facing changing environments.

## Results

### Wing size distribution

We sampled individuals in four population pairs inhabiting geographically close tidal and seasonally inundated habitats in Belgium (Be; 48 ind.), France (Fr; 48 ind.), Portugal (Po; 16 ind.) and Spain (Sp; 16 ind.), as well as a tidal marsh population in the UK (Uk; 8 ind.) and a seasonally inundated habitat at the Mediterranean coast of France (Me; 8 ind.) (Fig 2A). Individuals from the seasonally inundated habitats had significantly longer wings and larger body sizes compared to those from the tidally inundated marshes (*F*_1,117_ = 1904.4, *P* < 0.0001 for wing length and *F*_1,117_ = 162.29, *P* < 0.0001 for body size). The degree of divergence in wing length between the two ecotypes varied among the four population pairs (*F*_3,117_ = 23.11, *P* < 0.0001), with highly divergent wing lengths in Sp and Po, and some overlap in wing lengths in Be and Fr. Based on these clear-cut differences in wing length, we refer to the populations sampled in the tidal or seasonally inundated habitats as belonging to the short-winged (S) tidal or long-winged (L) seasonal ecotype, respectively.

### Population structure and genome wide divergence and diversity

RAD-tag sequences filtered for a minimum coverage of 10 and quality score higher than 20 resulted in 27,757 SNPs with an average individual depth of 62.9 (± 51 std). Of these, 10,052 SNPs distributed over 1,142 RAD-tag loci were present in at least 80% of the individuals and were used in further analysis. Average nucleotide diversity (*π*) at RAD-tags did not differ between ecotypes (GLMM with RAD-tag ID as random effect: Ecotype effect: *F*_1, 1977_ = 0.31, *P* = 0.6), but differed among population pairs (Population effect: *F*_3, 1977_ = 11.5, *P* < 0.0001, S1 Table). The most southern populations had a significantly higher nucleotide diversity compared to the northern populations. The difference in nucleotide diversity among population pairs was also consistent among ecotypes (Population*Ecotype interaction: *F*_3, 1977_ = 2.2, *P* = 0.09).

**Fig 3:**
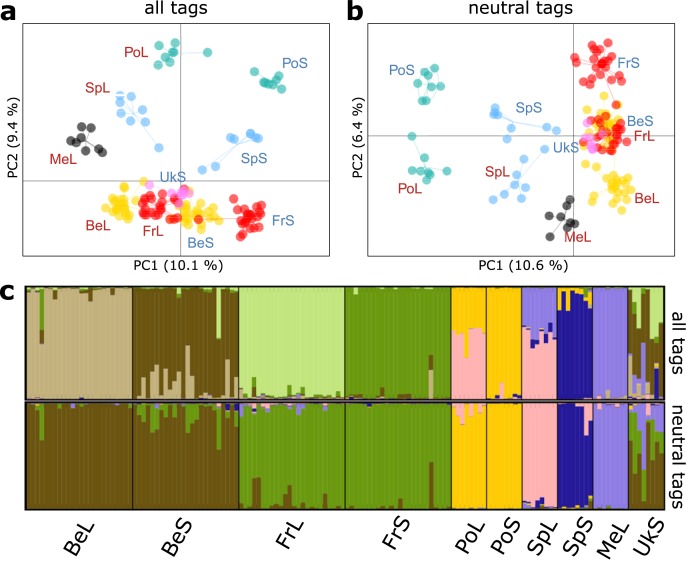
Population structure among the studied *Pogonus chalceus* populations. **(a.)** Principal Coordinate Analysis (PCoA) for sequenced samples using all RAD-tags. **(b.)** PCoA for sequenced samples when restricting the SNPs to a ‘neutral’ set wherein we excluded RAD-tags containing a SNP with a signature of divergent selection **(c.)** Population structure of the ten *P*. *chalceus* populations based on Bayesian clustering [32]. The best supported number of clusters was 8 for all RAD-tags and 6 for neutral RAD-tags (see S1 Fig). See Fig 1 for population codes.

Genetic differentiation (*F*_*ST*_) among the 10 different populations, varied considerably and ranged from a low (BeS vs. UkS: *F*_*ST*_ = 0.052) to a high degree of differentiation (PoS vs. MeL: *F*_*ST*_ = 0.37) (S1 Table). Principal Coordinate Analysis (PCoA) using all SNP data divided samples largely according to ecotype along the first PCo axis, whereas the second PCo axis grouped samples according to geographic location (Fig 3A). When restricting the SNPs to a ‘neutral’ set wherein we excluded RAD-tags containing a SNP with a signature of divergent selection (see *Outlier loci*), the importance of both axes was reversed with the first axis ordinating populations according to their geographic location rather than by ecotype (Fig 3B). Genetic differentiation increased significantly with increasing geographic distance between the populations (*r*_*S*_ = 0.37, *P* = 0.017) and was higher when populations belonged to a different ecotype (*r*_*S*_ = 0.33, *P* = 0.02). For the ‘neutral’ set there was an even stronger effect of geographic distance on genetic differentiation (*r*_*S*_ = 0.54, *P* = 0.002), while the significant ecotype effect disappeared (*r*_*S*_ = 0.09, *P* = 0.2). Bayesian clustering [32] of individuals based on their genotypes supported 8 and 6 genetically distinct populations (*K*) for the ‘total’ and ‘neutral’ SNP set, respectively (Fig 3C; S1 Fig). For the ‘neutral’ SNP set, individuals from the same population pair (except Sp) clustered together as a single population, irrespective of their ecotype.

### Demographic reconstruction

**Fig 4:**
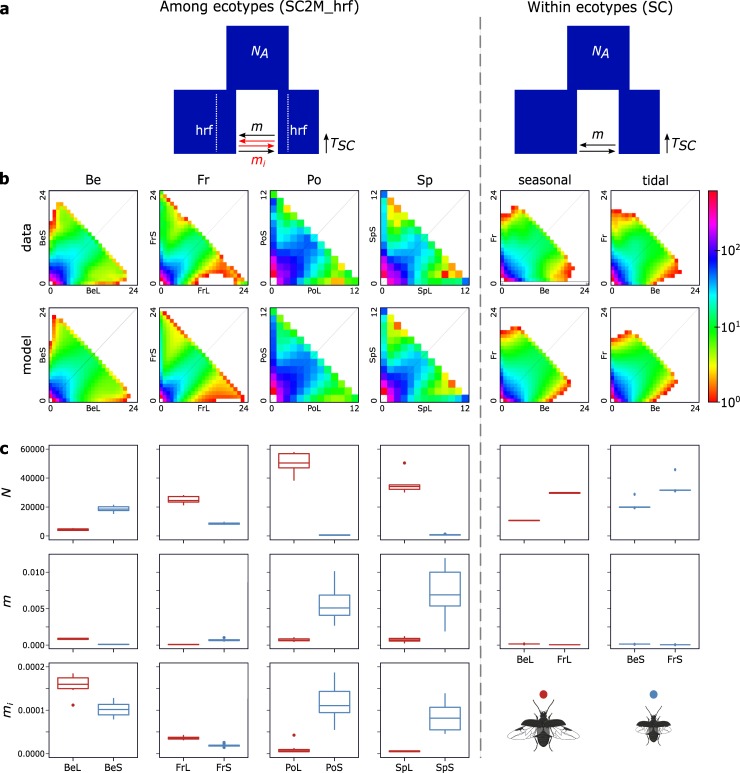
Demographic parameter estimates for population pairs. **(a.)** The assumed demographic model that best fit the data is a secondary contact model with heterogeneous gene flow and heterogeneous population size due to the effect of linked selection (SC2M_hrf) for the ecotypic population pairs and a secondary contact model with homogeneous gene flow (SC) for the within ecotype population pair comparisons (see S2 Table for details on model fitting). **(b.)** Data (first row) and model (second row) based joint allele frequency spectra (JAFS) of the ecologically diverged pairs Be, Fr, Po and Sp and the within ecotype population pair comparisons. JAFS are projected to 24 individuals, except for the populations Po and Sp where the JAFS was projected to 12 individuals. **(c.)** box-and-whisker plots of the estimated population size (population mutation rate theta) and effective number of migrated gene copies per generation into each ecotype of the inferred neutral (*m*) and non-neutral (*m*_*i*_) part of the genome.

We inferred the demographic history of divergence for each population pair using the joint allele frequency spectrum (JAFS) as implemented in *δ*a*δ*i [33]. In all four ecotypic population pairs the JAFS showed a pattern wherein most alleles were present in comparable frequencies in both populations (high density at the diagonal of the JAFS; Fig 4). However, at the same time the JAFS showed an increase in frequency of alleles present at very low frequencies in either one of the two populations but at high frequencies in the opposite population (high densities towards the upper left and lower right corner of the JAFS; Fig 4). This was particularly the case for the short-winged tidal populations from Fr, Po and Sp, in which we observed a relatively high frequency of alleles that were present in very low frequency in the long-winged seasonal ecotype, but nearly reached fixation in the short-winged tidal ecotype. These patterns contrasted sharply with those present in the JAFS of the within ecotype comparison of geographically separated populations. Here, a lower density of alleles was observed both for alleles that were present in comparable frequencies, as well as for alleles with highly profound frequency differences (Fig 4).

In all four among ecotype pair comparisons, demographic models incorporating gene-flow after the divergence (IM and SC) and heterogeneous genomic divergence (“2M”) and/or heterogeneous population size (“hrf”) were clearly better supported compared to models that did not incorporate these effects. A Secondary Contact (SC) model incorporating both heterogeneous gene-flow and population size (SC2M_hrf) yielded the best fit for all ecotype comparisons and predicted the observed JAFS reasonably well (Fig 4 and S2 Fig). However, this fit was only marginally better than a Secondary Contact model with heterogenous genomic divergence, but without heterogeneous population size (SC2M) for the population pairs Fr and Po and an Isolation-with-Migration model with heterogeneous genomic divergence (IM2M) for population pair Sp.

We based interpretation of the estimates of the demographic parameters on the SC2M_hrf model for all four ecotypic population pair comparisons. Estimates of the effective population size revealed a distinct pattern in the relative population sizes of the two ecotypes. In the northern population pair Be, the population size of the short-winged tidal ecotype was estimated to be around four to five times larger compared to the population size of the long-winged seasonal ecotype. In contrast, towards more southern latitudes, this pattern was reversed with population sizes of the short-winged ecotype being estimated to be nearly 50 (Sp) to 100 (Po) times smaller compared to those of the long-winged seasonal ecotype (Fig 4). Population migration rates (*M*) were strongly related to the ecotypic differences in population size and a general trend was observed of higher migration rates from the ecotype with the largest population size towards the ecotype with the smallest population size. These migration rates were substantial and varied from approximately 1.5 gene copies per generation for both Be and Fr up to more than 30 gene copies per generation for Po and Sp, respectively.

The estimated proportion of the genome showing restricted gene flow between both ecotypes was comparable among the four population pairs and varied between 27% and 33% of the genome. The reduction in effective migration rate of this part of the genome was stronger for the southern population pairs Po and Sp (reduction of 97.6% and 99% of the neutral migration rate, respectively), compared to the northern populations Fr and Be, with a respective reduction of 78% to 31% of the neutral migration rate. Initial divergence times between the ecotypes were estimated between 43 Kya (Be), 64 Kya (Po) and more than 100 Kya (Fr and Sp), while the onset of secondary contact was estimated between 1,6 Kya (Be) to 23 Kya (Fr).

The results obtained from the among ecotype pair comparisons were in clear contrast with the within ecotype comparisons (Be and Fr only). Here, models assuming homogeneous genomic divergence were equally well supported as models assuming heterogeneous genomic divergence (S2 Fig). Fit of the IM and SC model, either with or without heterogeneous population size, was comparable for the geographically separated long-winged seasonal populations, while the SC model was better supported for the geographically separated short-winged tidal populations. The estimated migration rates were substantially lower compared to those of the among ecotype comparisons and were estimated to be higher from Fr into Be compared to the opposite direction (Fig 4).

### Outlier loci

**Fig 5:**
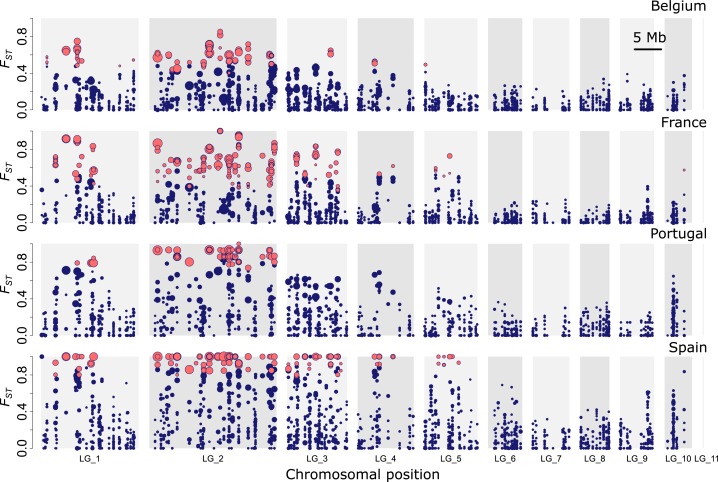
Genomic divergence (*F*_*ST*_*)* between *Pogonus chalceus* ecotype pairs. Outlier SNPs within each population pair, as identified by BayeScan [34], are indicated in red. Size of points is proportional to the log_10_BF reported by BayEnv2 [35] and indicates the degree of support that allele frequencies are significantly correlated with habitat type across all sampled populations. Markers on LG10 are significantly sex-linked. Average *F*_*ST*_ values across SNPs between all population comparisons are reported in S1 Table and the distribution of *F*_*ST*_ values is given in S3 Fig.

Despite the apparent close genetic relationship of long- and short-winged ecotypes within each geographic population pair (S1 Table), we observed substantial heterogeneity in *F*_*ST*_ across SNPs (Fig 5, S3 Fig). A substantial number of SNPs showed *F*_*ST*_ values that exceeded 0.5 in the ecotype comparisons. For some of these SNPs, different alleles even reached almost complete fixation in the different ecotypes. This proportion of SNPs with *F*_*ST*_ values higher than 0.5 increased towards the more southern population pairs (Be: 2.1%, Fr: 4.5%, Po: 6.3% and Sp: 11.1%). In contrast, only very few *F*_*ST*_ values exceeded 0.5 when similar ecotypes were compared from different population pairs (e.g. Be versus Fr; S3 Fig). SNPs that were strongly differentiated in one particular population pair were also significantly more differentiated in any of the other population pairs (0.498 < *r* < 0.66; *P* all < 0.0001; S3 Fig), providing support for extensive sharing of highly differentiated SNPs among population pairs.

Significant outliers were identified using two approaches; BayeScan [34] to identify outliers from the genome wide background within each population pair and BayEnv2 [35] for associations between SNP allele frequencies and habitat type (coded as -1 or 1 if tidal or seasonal habitat, respectively) across all populations. BayeScan identified a total of 512 (3.2%) SNPs that were clustered on 109 (15%) assembled RAD-tag loci with stronger differentiation as expected by chance in at least one of the ecotype comparisons (false discovery rate = 0.05; i.e. on average 4.70 outlier SNPs per RAD-tag locus). BayEnv2 identified a total of 75 (0.48%) SNPs in 32 (6.3%) assembled RAD-tag loci having allele frequencies that were strongly associated with the ecotypic divergence across all investigated populations (log_10_BF = 4). On average 75% of these SNPs were identified as significant outliers with BayeScan. Despite this general agreement in SNPs that were consistently identified by both approaches, few SNPs that were strongly supported to be outlier SNPs across the entire range (BayEnv2; log_10_BF > 4) were not significantly differentiated within some regional ecotype comparisons. Conversely, significant outliers at the regional level were sometimes not supported to be outliers across the entire range and, therefore, likely population specific (S4 Fig and S5 Fig). SNPs were mapped to a genome assembly (S1 Supporting Methods), which revealed that SNPs with a high *F*_*ST*_ value clustered into several unlinked regions that were distributed over a large proportion of the genome (Fig 5). These regions with outlier SNPs were largely consistent across the different population pairs and are primarily clustered on the first half of LG_1, across the full length of LG_2 and LG_3 and at the center of LG_4 (Fig 5). R-squared values between the allele frequencies at outlier loci show a sharp decline with distance between the considered loci within the linkage groups (S6 Fig). This suggests the absence of, at least large, structural chromosomal rearrangements for explaining the observed divergence patterns (i.e. divergent allele combinations recombine). No outlier SNPs were observed on LG_6 to LG_10. Yet, some more subtle differences could be observed as exemplified by the central region of LG_5 where high genomic differentiation was only observed for the Fr and Sp population pair, but not in the Be and Po population comparison. The nuclear-encoded mitochondrial NADP^+^-dependent isocitrate dehydrogenase (*mtIdh*) locus, that was previously identified to be strongly associated with the ecotypic divergence [27,28,30,36], is located approximately in the middle of LG_2 (scaffold Pchal00589: 148,569–150,947, chromosome LG_2: 9,802,630–9,805,108). This genomic region includes several other outlier RAD-tag loci and suggests *mtIdh* may not be a direct target of selection, but rather linked to other divergently selected loci.

### Sequence variation and phylogenetic reconstruction at outlier loci

**Fig 6:**
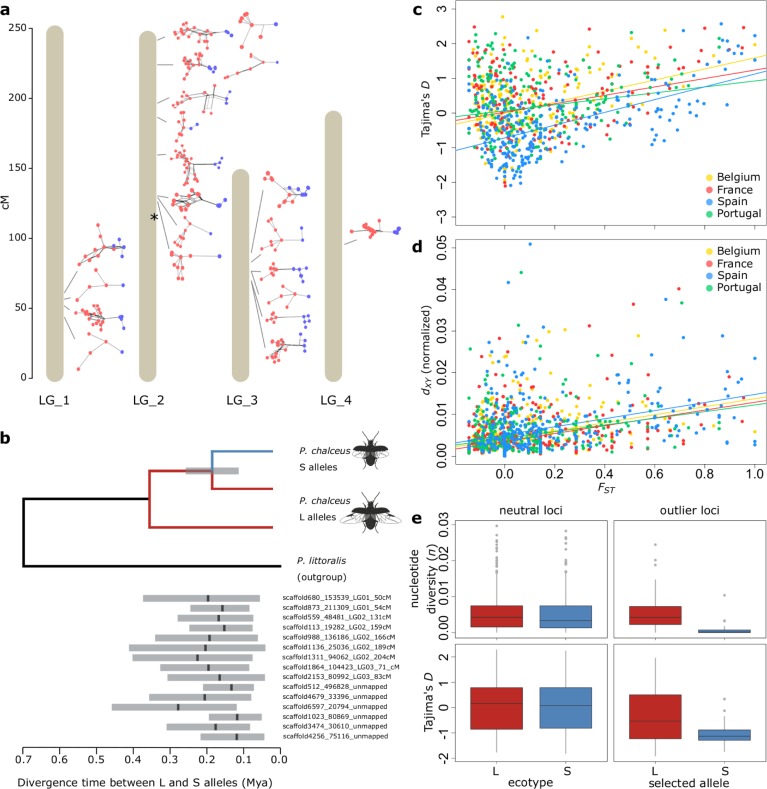
Haplotype structure and diversity at divergently selected loci. **(a.)** Haplotype networks of RAD-tags containing outlier SNPs and at least 10 variable sites (BayEnv2; log10BF > 4) at the different linkage groups. Haplotypes selected in short-winged populations are depicted in blue, haplotypes selected in long-winged populations are depicted in red. The asterisk indicates the position of the *mtIdh* gene studied in [36] **(b.)** Estimated divergence time (Mya) between alleles selected in short-winged (blue) versus long-winged (red) populations. The tree represents the general phylogenetic relationship between short- and long-wing selected alleles and the estimated divergence point. **(c.)** Relationship between *F*_*ST*_ and Tajima’s *D* (considering both ecotypes for each population) and **(d.)** absolute nucleotide divergence, *d*_*XY*_, scaled relative to the divergence from the outgroup species *Pogonus littoralis* in the four population pairs. **(e.)** Comparison of nucleotide diversity (π) and Tajima’s *D* at neutral loci of long-winged (L) and short-winged (S) populations (left) and between haplotypes at outlier RAD-tags selected in L or S populations (right).

Haplotype networks and trees of the 1.2 kb sequence alignments obtained from RAD-tag loci with an outlier SNP (BayEnv2 [35]; log_10_BF > 4) show that haplotypes selected in short-winged tidal populations are derived and generally clustered as a strongly supported monophyletic clade of closely related sequences (clade support level > 0.96; Fig 6A and 6B and S5 Fig). This clustering supports a singular mutational origin of alleles selected in the short-winged tidal ecotype at each of the investigated outlier tags. These alleles appeared to be derived as they most frequently constituted a subclade within those selected in the long-winged seasonal ecotype (S7 Fig). This is in line with the observation that all other species within the genus *Pogonus* are long-winged [37]. The average absolute divergence between the differentially selected haplotypes (*d*_*XY*_ = 0.011 ± 0.0014) was about 1.65 times higher compared to the average divergence between two randomly chosen haplotypes at these loci (*π*_tot, outliers_ = 0.0067 ± 0.00097, *t*-test: *P* < 0.0001) and highlights a deep divergence between the alleles that are differentially selected between both ecotypes. Dating the divergence time between these allelic clusters using BEAST [38] and the divergence from *P*. *littoralis* as a calibration point (620 Kya [36]), pointed towards comparable divergence times across outlier loci (Fig 6B and S5 Fig). The divergence time of the alleles selected in the short-winged tidal ecotype ranged between 120 Kya and 280 Kya, with an average of 189 Kya ± 90 Kya and suggests that the divergence took place during the Late Pleistocene.

Sequences from more strongly differentiated RAD-tags had a significantly higher Tajima’s *D* (pooled across ecotypes within each population pair; *F* = 57.36, *P* < 0.0001; Fig 6C) and absolute nucleotide divergence between the ecotypes (normalized by the divergence from the outgroup *P*. *littoralis* = *d*_*XY*_ */ d*_*XY*, *P*. *littoralis*_, see Methods for details; *F*_*ST*_: *F* = 83.9, *P* < 0.0001; Fig 6D). The significant relation between *F*_*ST*_ and absolute divergence (normalized *d*_*XY*_) further supports that the observed heterogeneity in genomic divergence between the ecotypes is the result of divergent selection of ancient alleles embedded within a genome that is homogenized between the ecotypes, rather than selection on recently obtained new mutations [39,40]. Furthermore, a reduced recombination rate was observed between haplotypes that are divergently selected between long- and short-winged populations (*r*^2^ = 0.140) compared to the recombination rate observed within populations (*r*^2^ = 0.184).

We further observed that nucleotide diversity (*π*) of haplotypes associated with the short-winged tidal ecotype was strongly reduced and tended to be nearly seven times lower (*π*_S_ = 0.0009 ± 0.0003) compared to those associated with the long-winged seasonal ecotype (*π*_L_ = 0.0062 ± 0.0003) (GLMM with tagID as random effect: Ecotype effect: *F*_1, 66_ = 25.12, *P* < 0.0001; Fig 6E). This difference was consistent among the four populations (Ecotype*Population interaction: *F*_3, 64_ = 0.87; *P* = 0.45). In contrast, nucleotide diversity at RAD-tags showing no elevated levels of divergence between the ecotypes was comparable between both ecotypes (GLMM with RAD-tag as random effect: Ecotype effect: *F*_1, 599_ = 0.98, *P* < 0.3; Fig 6E). The nucleotide diversity of the haplotypes associated with the long-winged seasonal ecotype was also comparable to average nucleotide diversity observed at non-outlier loci (*π*_tot, neutral RAD-tags_ = 0.0057 ± 0.0003), showing that only haplotypes associated with the short-winged tidal ecotype have this reduced nucleotide diversity (Fig 6E). Similarly, Tajima’s *D* of haplotypes associated with the short-winged tidal ecotype was significantly lower compared to those of the long-winged seasonal ecotype (F_*1*,*27*_ = 11.7; *P* = 0.002; Fig 6E) and suggests a recent spread of alleles of the short-winged tidal ecotype along the Atlantic European coast.

### Quantifying standing genetic variation

**Fig 7:**
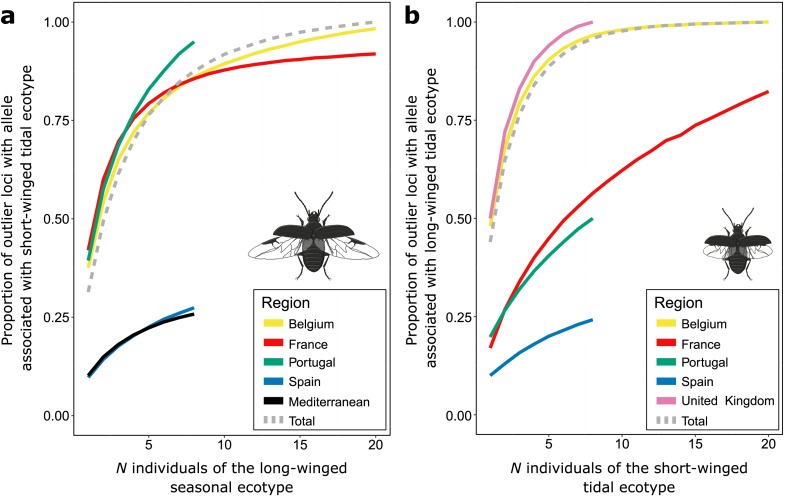
Quantifying standing genetic variation. Accumulation curves of **(a.)** the proportion of outlier loci containing at least one copy of the allele associated with the short-winged tidal ecotype in a random sample of *N* individuals of the long-winged seasonal ecotype and **(b.)** the proportion of outlier loci containing at least one copy of the allele associated with the long-winged seasonal ecotype in a random sample of *N* individuals of the short-winged tidal ecotype. Proportions are averaged over 100 replicates of *N* individuals.

Here, we quantify the extent to which polymorphism at outlier loci determines the genetic variation of each ecotype to potentially adapt to the alternative environment. More specifically, we calculated how many individuals does one need to sample to capture most of the genetic variants that are selected in the alternative environment? The outlier analyses revealed that restricted regions within the genome are significantly more diverged as expected by chance and thus likely linked to sites under divergent selection, but generally did not reach fixation in most of the investigated population pairs (Fig 5). This was also indicated by the reconstruction of the demographic history, which revealed that admixture between the ecotypes also involves genomic islands. Therefore, we calculated the frequency of alleles that are selected for in the alternative habitat at outlier loci for each ecotype. We focused on SNPs whose allele frequencies were strongly associated with the ecotypic divergence across all investigated populations (BayEnv2[35]; log_10_BF = 4). If multiple outlier SNPs were situated on the same RAD-tag, only the most strongly supported SNP was selected. Individuals of the long-winged seasonal ecotype contained on average at 10% (SpL) to 42% (BeL) of the outlier loci at least one allele associated with the alternative, short-winged tidal ecotype. Similarly, individuals from the short-winged tidal ecotype contained alleles associated with the long-winged seasonal ecotype at 10% (SpS) to 48% (BeS) of the outlier SNPs. Moreover, random sampling of an increasing number of individuals showed a steep increase in the proportion of outlier SNPs with at least one allele associated with the alternative habitat (Fig 7). For example, a random sample of only eight individuals of the long-winged seasonal ecotype of Be, Fr and Po contained at least one copy of the allele selected in the short-winged tidal ecotype at more than 80% of the outlier loci (Fig 7A). This demonstrates that different individuals from the same population generally carry alleles that are selected in the alternative habitat at different loci and suggests the presence of substantial standing genetic variation in individuals sampled in the seasonally inundated marshes to adapt to tidal marshes. Only for the most southern long-winged seasonal populations (SpL and MeL), outlier loci that are likely linked to alleles associated with the short-winged tidal ecotype are present at lower frequencies and these populations are unlikely to contain the full set of alleles associated with the short-winged ecotype. For FrS, PoS and particularly SpS, long-wing selected alleles accumulated at a much lower rate under random sampling of individuals of the short-winged tidal ecotype (Fig 7B).

## Discussion

Understanding the genomic basis of repeated and fast ecological adaptation provides unique insights into the process of evolutionary diversification [14,15,17]. While evidence is accumulating that cases of repeated adaptation are largely driven by selection on standing genetic variation [41], the evolutionary origin of this variation generally remains less well characterized [21,22].

In *P*. *chalceus*, several unique observations help to disentangle the complex history of fast and parallel ecological divergence. We found that most loci with elevated levels of divergence between ecotypic pairs had identical or closely related haplotypes within the tidal populations. This strongly agrees with scenarios in which differentiation between the ecotypes is based on selection of the same alleles throughout the species’ range. Hence, the parallel ecotypic divergence in *P*. *chalceus* has evolved from standing genetic variation rather than through selection of alleles that arose by *de novo* mutations within each region (Figs 1A and S1). Further, the estimated time at which the alleles associated with each ecotype diverged, as well as their nucleotide diversity patterns, appeared highly consistent across these loci. This genealogical consistency at unlinked loci would not be expected if the alleles associated with the tidal ecotype arose by mutations within the ancestral long-winged seasonal ecotype (Figs 1B and S2). Instead, the shared evolutionary history at these unlinked genomic regions is in line with a singular evolutionary origin of the short-winged tidal ecotype (Figs 1C, 1D, S3 and S4). Together with the deep divergence between alleles associated with the tidal populations compared to alleles associated with the seasonal populations, this suggests that the tidal alleles evolved, at least partly, in geographic isolation.

After the initial divergence of the tidal and seasonal ecotypes, gene flow at loci within the genomic islands of divergence has likely resulted in the highly polymorphic populations of Po, Fr, Be and Uk. In *P*. *chalceus*, this high rate of polymorphism within populations at loci with elevated divergence between ecotypic population pairs partially obscures the distinctness of the ecotypes at both the genetic and phenotypic level. For example, wing sizes of beetles from the tidal populations Be showed some overlap with the wing size of the seasonal populations of Be, Fr and Po. This high rate of polymorphism at outlier loci complicates distinguishing between a secondary contact model (Figs 1C and S3) versus a scenario of *in-situ* divergence by selection of introgressed alleles (Figs 1D and S4), because distinguishing these depends on the proportion of “tidal alleles” in individuals that colonize tidal habitats and vice versa. This proportion may range from very high, in which individuals are nearly pure short-winged (Fig 1C and S3), to very low, in which dispersing individuals are nearly pure long-winged in allelic composition with few short-winged alleles (Figs 1D and S4).

Demographic modelling of the population divergence with *δ*a*δ*i showed that population divergence conforms best to a secondary contact model (SC), which points towards a signature of geographic isolation between the ecotypes in the JAFS. The timing of the initial split between the ecotypes in this secondary contact model was estimated at ~50 to ~100 Kya, depending on the population pair and likely refers to the initial divergence between the ecotypes. This timing of the initial divergence inferred by *δ*a*δ*i was more recent than the estimated divergence time between the differentially selected alleles as estimated by BEAST (~190 Kya). The more recent times obtained for the ecotype divergence by *δ*a*δ*i are likely attributed to the estimation of population divergence rather than estimation of the time at which the differentially selected alleles coalesce, as is the case in the molecular dating approach at outlier loci. Further, the inferred low levels of differentiation between ecotype pairs at neutral loci by *δ*a*δ*i suggest considerable admixture after the initial divergence of these ecotypes. More precisely, demographic reconstruction estimated gene-flow levels at neutral loci in the order of 1.4 (0.007%) to 44.2 (0.5%) gene copies per generation within the last 1,6 to 23 Kya, which are sufficient to swamp the initial neutral genetic differences between the ecotypes [42]. This is particularly illustrated by the current lower neutral differentiation between ecotypes from the same region compared to the differentiation within ecotypes between regions. A consequence of the high rates of gene flow, combined with selection on ancient adaptive alleles that evolved in allopatry imply that our demographic analysis does not allow to discriminate among scenario’s S3 and S4 as both are expected to result in highly similar JAFS spectra. As the demographic scenarios do not explicitly incorporate selection, it remains difficult to discriminate if the genomic islands involve genomic regions that are resistant to introgression after secondary contact, or rather the result of differential selection on alleles that evolved in isolation within an otherwise genetically homogeneous population.

Despite the difficulty of differentiating a secondary contact model from a scenario with recent *in-situ* divergence by selection of introgressed alleles, several observations support that the current distribution of the ecotypes likely involves the recent and repeated *in-situ* evolution of short-winged tidal populations (Figs 1D and S4). First, the high levels of admixture results in polymorphism at genomic islands of divergence and increases the potential of populations to easily adapt to the alternative environmental conditions. Indeed, quantifying the amount of short-winged tidal selected alleles present in long-winged seasonal populations revealed that more than 80% of the alleles associated with the short-winged tidal ecotype are present in a random subset of between 5 and 15 individuals of the long-winged seasonal ecotype. Thus, genetic constraints for the evolution of the short-winged ecotype out of long-winged individuals, and vice versa, appear to be surprisingly low. Second, short-winged individuals are unable to disperse by flight between the currently highly isolated salt-marsh areas. The fragmented distribution of tidal salt marshes along the Atlantic coast renders it therefore unlikely that they were colonized by short-winged individuals based on terrestrial dispersal alone, in particular because the species strongly avoids unsuitable habitat patches [37]. Direct support for this mechanism is found in the isolated tidal population “Baai van Heist” (Be, not included in the current study) wherein we observed a gradual evolution towards smaller wings after colonization by a long-winged founder population (S1 Supporting Results). Third, we previously put forward a behavioral mechanism that may explain the spatial sorting of these ecotypes into their respective habitats (i.e. long-winged beetles tend to avoid frequent flooding in tidal habitats, whereas short-winged beetles stay submerged during short tidal flooding events), which may reduce gene flow and induce rapid divergence of the genetically distinct ecotypes within a sympatric mosaic [29].

Major geographic expansions and contractions of the tidal and seasonal habitat types have likely occurred since the initial divergence of the short-winged tidal ecotype. We estimated the evolution of the short-winged tidal associated alleles to have occurred about 190 Kya, which corresponds to the Mid to Late Pleistocene. Since then, Europe has been subject to at least one interglacial (130–115 Kya) and one glacial (115–12 Kya) period. These major climatic changes fragmented the Euro-Atlantic coastline, potentially creating opportunities for the initial evolution of the short-winged tidal ecotype in the partially isolated large coastal floodplains that extended, for instance, around the North-Sea basin [43]. Due to these glacial oscillations and more recent admixture between ecotypes, reconstructing the historic distribution of the initial short-winged population is at present difficult. However, during the last glacial maximum a short-winged tidal refuge population was likely located more southward relative to the current species distribution, as it is deemed unlikely that the species persisted at the current northern latitudes of its distribution [37]. Increase in temperature after the last glacial maximum resulted in the re-development of large coastal floodplains at northern latitudes [43] and likely led to a northwards expansion of the species. The onset of admixture between the ecotypes estimated between 1.5 Kya and 23 Kya ago, coincides with this period. It seems therefore plausible that both ecotypes came into secondary contact during the northward expansion. The lower degree of overall genetic differentiation between the ecotypes in the more northern population pairs Be and Fr, less profound phenotypic differentiation and lower overall genetic diversity (*π*) are all consistent with a northward expansion of an admixed population and more recent ecotypic divergence. Similar findings of a decrease in divergence towards more northern latitudes that support shorter divergence times in the north have also been observed in parallel ecotypes of lampreys [44]. Potentially, this expansion may have further facilitated the maintenance of deleterious short-winged tidal selected alleles in the expanding long-winged seasonal population [45], which then spread quickly in the emergent tidal coastal floodplains. The low nucleotide diversity and significantly lower Tajima’s D of the haplotypes associated with the short-winged tidal ecotype further agree with the rapid and recent spread of alleles associated with tidal ecotype.

The two-step process of initial divergence in an ancient and potentially isolated population and subsequent admixture putatively also applies to other examples of fast and repeated ecological divergence. Repeated ecological divergence at the same loci has been reported in some iconic examples of parallel evolution, such as stickleback, cichlid fishes and *Heliconius* butterflies [46–51]. These loci have in many cases been assigned to shared ancient polymorphisms that were present in the population before the evolution of the currently observed divergent populations [51]. Moreover, many of these loci are sometimes identified and are unlinked throughout the genome, such as in fruit flies, *Timema* walking sticks and *Littorina* sea snails [7,52,53]. The genetic signature of the evolution of the *P*. *chalceus* ecotypes shows strong analogies to these well-studied cases of repeated adaptation. In cichlid fishes, moreover, it has been extensively argued that divergence in isolation and subsequent admixture may have provided the genetic material for the incredibly diverse and recent adaptive radiations of cichlid fish [11,54]. Untangling the evolutionary history of the alleles involved in these and other cases will help in better understanding the processes that drive parallel divergence as well as fast responses to environmental change.

### Conclusion

The initial evolution of co-adapted alleles at multiple physically unlinked loci is facilitated in geographic isolation [3]. Subsequent admixture of gene pools may then enrich the adaptive genetic variation and allow for subsequent fast and repeated adaptation. In agreement to this, in *P*. *chalceus* populations the alleles required to adapt to the alternative environment are found to be maintained in the source population. These loci are expected to be maladaptive within the source population and it is likely both the temporal and spatial repetition of this divergence, combined with relatively high levels of gene flow and range expansion, that maintain these allele frequencies. Glacial cycles, in particular, can be expected to have played an important role in this process. During glacial cycles, episodes of fission and fusion of the different ecotypes may have generated strong opportunities for both the evolution of adaptive genetic variants as well as the maintenance of genetic polymorphisms by admixture [55]. As exemplified by the evolution of the *P*. *chalceus* ecotypes, historic selection pressures could therefore play a pivotal role in determining the rate, direction and probability of contemporary adaptation to changing environmental conditions. The proposed mechanism illustrates that the distinction between *in-situ* divergence and secondary contact is less clear-cut as generally assumed if populations are highly admixed and that, moreover, both processes can be involved at different time frames. An important implication is that this mechanism might reconcile different views on the geography of ecological divergence in which adaptive divergence between closely related populations is either interpreted as primary divergence, and thus the onset of speciation [2], or the result of secondary introgression after initial ecological divergence in allopatry [22,25,56].

## Methods

### Sampling

Diverged population pairs of *P*. *chalceus* were collected from both tidal and seasonal salt marshes extending nearly the entire species range (Fig 2) [37]. We sampled four geographically isolated population pairs (separated between approximately 450 km and 900 km) of a tidal and seasonally flooded inland population each. Wing and elytral sizes were measured by means of a calibrated ocular with a stereomicroscope. We further conducted RAD-seq genotyping on two specimens of the long-winged outgroup species *P*. *littoralis*, which were sampled in the Axion Delta, Thessaloniki, Greece (S3 Table).

### RAD-tag sequencing

DNA was extracted using the DNA extraction NucleoSpin Tissue kit (Macherey-Nagel GmBH). Extracted genomic DNA was normalized to a concentration of 7.14 ng/μl and processed into RAD libraries according to Etter *et al*. (2011), using the restriction enzyme SbfI-HF (NEB) [57]. Final enrichment was based on 16 PCR cycles. A total of nine RAD libraries including 16 individuals each and, hence, a total of 144 individuals were sequenced paired-end for 100 cycles (i.e. 100 bp) in a single lane on an Illumina HiSeq2000 platform according to manufacturer’s instructions. The outgroup *P*. *littoralis* specimens were sequenced separately. The raw data was demultiplexed to recover individual samples from the Illumina libraries using the *process_radtags* module in Stacks v1.20 software [58]. Reads were quality filtered when they contained 15 bp windows of mean Phred scores lower than 10. PCR duplicates were identified as almost (i.e. allowing for sequencing errors) identical reverse read sequences and removed, using a custom Perl script [59].

### Genome assembly

Total DNA was extracted from individuals captured in the canal habitat of the salt marshes in the Guérande region (France), using the DNA extraction NucleoSpin Tissue kit (Macherey-Nagel GmBH). Illumina paired-end (100 bp) and mate-paired (49 bp) libraries were constructed with insert sizes of 200 bp, 500 bp, 800 bp, 2 kb and 5 kb and sequenced on an Illumina HiSeq2000 system according to the manufacturer’s protocol (Illumina Inc.). Adapter contamination in reads was deleted using Cutadapt v1.4 [60] and reads that did not have a matching pair after adaptor filtering were removed. Reads were corrected for sequencing error with SOAPec v2.02 [61], using a *k*-mer size of 17 and a low frequency cutoff of consecutive *k*-mer of 3. Sequencing of the 200 bp, 500 bp, 800 bp, 2 kb and 5 kb insert libraries resulted in a total of ~57.7 Gb of sequencing data, of which 56.6 Gb was retained after data cleaning (S4 Table). Reads were assembled using SOAPdenovo2 [61] using a *k*-mer parameter of 47, which was selected for producing the largest contig and scaffold N50 size after testing a range of *k*-mer settings between 19 and 71. The short insert libraries were used for both contig building and scaffolding. The long insert libraries were only used for scaffolding. SOAPdenovo GapCloser v1.12 tool [61] was used with default settings to close gaps emerging during scaffolding. We used DeconSeq v0.4.3[62] to identify and remove possible human, bacterial and viral contamination in the assembly (S5 Table). Completeness of the assembled genome was assessed by comparing the assembly with a dataset of highly conserved core genes that occur in a wide range of eukaryotes using the CEGMA pipeline v2.5 [63].

### Linkage map

To position the genomic scaffolds into linkage groups, we constructed a linkage map by genotyping parents and offspring (RAD-seq) from four families (S6 Table). For the parental generation, we used lab-bred individuals (F0) whose parents originated from the French population, to ensure that they had not been mated in the field. A total of 72 F1 offspring (*n* = 23, 14, 23 and 12 offspring from each family) were raised till adulthood and subsequently genotyped, together with their parents. To maximize the number of scaffolds comprising a marker, RAD-tag sequencing was based on a *Pst*I-HF (NEB) digest (6 bp recognition site) instead of the SbfI-HF (NEB) digest (8bp recognition site) of the population genomic analysis. Final enrichment was based on 16 PCR cycles. Illumina HiSeq sequencing resulted in a total of 237 M paired-end reads, of which 113 M remained after quality filtering and removal of PCR duplicates. Reads were mapped to the draft reference genome with BWA-mem [64] using default settings. Linkage map reconstruction was performed with LepMAP2[65]. LepMAP2 reconstructs linkage maps based on a large number of markers and accounts for lack of recombination in males due to achiasmatic meiosis, which is suggested in *P*. *chalceus* and male Caraboidea in general [66] (S1 Supporting Methods).

### Population genomic analysis

Quality and clone filtered paired-end reads of the 144 field captured individuals were mapped to a draft reference genome with BWA-mem [64]. Indel realignment, SNP and indel calling was performed with GATK’s UnifiedGenotyper tool [67]. Paired-end sequencing of the approximately 200 to 600 bp RAD tag fragments adjacent to symmetric SbfI restriction sites allowed us to obtain sequence information of 1,200 bp fragments (paired RADtag) around each restriction site. Hence, after SNP calling we retained all sites within 1,200 bp windows around each SbfI recognition site in the genome, totaling 732,884 bp of sequence. Haplotype phasing was subsequently first performed with GATK ‘read-backed phasing’ [67], while the remaining unphased sequences were phased with Beagle v4.1 [68]. A reliable SNP set was then obtained by retaining only SNPs with genotype quality higher than 20, average depth higher than 10 and a minor allele frequency higher than 0.01 (more likely to result from genotyping errors) in at least 80% of the individuals.

### Analysis of population structure

Pairwise *F*_ST_-statistics [69] across RAD-tags were calculated for all pairwise population comparisons using Genepop v4.5.1 [70]. Principal Coordinate Analysis was performed using *adegenet* in R [71]. To minimize dependence due to physical linkage among SNPs, we randomly selected one single SNP per paired RAD-tag. The average degree of linkage disequilibrium among these SNPs was sufficiently low (R^2^ = 0.03) to consider them as independent loci. We also constructed a ‘neutral’ subset by excluding SNPs located on scaffolds showing signatures of divergent selection (20.5% of all SNPs). As a criterion, we excluded scaffolds containing a SNP with a log_10_(BF) > 3 as determined by BayEnv [72]. The Pearson correlation between genetic divergence and either geographic distance between the populations or ecotype (coded as 1 = different ecotype and 0 = identical ecotype) was assessed by a Mantel test in the vegan v2.2–1 package in R v3.1.3 [73]. Based on these two datasets, we used the Bayesian clustering algorithm implemented in STRUCTURE v2.3.4.[32] to assign individuals into *K* clusters based on their multilocus genotype. We applied an admixture model with three independent runs for each *K* = 2–10, 100,000 MCMC repetitions with a burn-in of 30,000, correlated allele frequencies among populations and no prior information on population origin. Default settings were used for the prior parameters. The best supported number of clusters (*K*) was determined from the increase in the natural logarithm of the likelihood of the data for different numbers of assumed populations.

### Demographic reconstruction of population divergence

We inferred the demographic history of divergence for each population pair by a diffusion approximation method as implemented in *δ*a*δ*i [33]. Given a particular demographic scenario, *δ*a*δ*i estimates the demographic parameters by comparing the expected with the observed joint allele frequency spectrum (JAFS). Demographic inference was conducted for the ecotypic population pairs Be, Fr, Po and Sp and within ecotypes for the populations Be and Fr. The JAFS was projected to 24 individuals for populations Be and Fr and to 12 individuals for the ecotypic population pairs of Po and Sp. We fitted three divergence scenarios, including a population split without subsequent gene-flow between the ecotype (Strict Isolation, SI), a split event followed by gene-flow (Isolation-with-Migration, IM) and a population split followed by a period of strict isolation and secondary contact afterwards (Secondary Contact, SC). Each model estimates the relative size of the two subpopulations compared to the size of the ancestral population (*v*_1_ and *v*_2_), the time of the split between the two subpopulations (*t*_S_) scaled by the ancestral population mutation rate, the rate at which migrants are exchanged into population *i* from population *j* (*M*_*i*←*j*_) and vice versa (IM and SC models only) and the time of secondary contact (*t*_SC_) (SC model only). Next, we incorporated heterogeneous genomic divergence to account for reduced gene flow in genomic regions associated with adaptive divergence (genomic islands) by estimating a proportion of the genome, *P*, with a reduced effective migration rate (*M*_(I),*i*←*j*_ and *M*_(I),*j*←*i*_) between the two subpopulations *i* and *j* (IM2M and SC2M) [74]. We further incorporated the effect of local reduction in *Ne* at neutral sites linked to sites subjected to positive or background selection by estimating a proportion *Q* with a population size reduced by a factor *hrf* [75].

We compared the fit of the different demographic models by means of the Akaike Information Criterium values (AIC = 2*k -*2*lnL*, with *k* the number of estimated parameters in each model and *lnL* being the logarithm of the likelihood of the model). After performing some preliminary runs to define appropriate parameter search spaces, we ran twenty replicated runs for each model and selected the five runs with the smallest AIC.

We obtained biologically more meaningful parameter estimates of the effective population sizes, migration proportions and splitting times by converting the mutation scaled estimates based on the mutation rate estimate, *μ* of *P*. *chalceus*. As an estimate for *μ*, we first selected all genomic sites (both variable and invariable) that are present with a minimal depth of at least 10 in all sequenced individuals of both *P*. *chalceus* and the outgroup species *P*. *littoralis*. Based on this SNP set, we obtained an average proportion of nucleotide differences between both species of 0.03587. The estimated divergence time between both species is 0.62 ± 0.06 Mya [36], yielding an estimated mutation rate of *μ* = (0.03587/2)/620,000 = 2.9*10^−8^ mutations/site/year. This mutation rate was used to calculate the effective population size, expressed as number of individuals, of the ancestral population *N*_*A*_ = *θ*_A_ /4*μL*, with *L* being the total sequence length from which SNPs were extracted in each population pair comparison. We subsequently obtained the subpopulation sizes as *N*_*i*_ = *v*_*i*_*N*_A_, the estimated divergence time (*T*_S_) and time at secondary contact (*T*_SC_) in years as *T*_S_ *=* 2*N*_A_ *t*_S_ and *T*_SC_ = 2*N*_A_ *t*_SC_, respectively, and the proportion of received migrant copies into population *i* from population *j* as *m*_*i*←*j*_ = *M*_*i*←*j*_/2*N*_A_ and in the opposite direction.

### Outlier loci detection

Support for loci showing significantly higher degrees of differentiation was first detected with BayeScan2.1 [34] within each population pair (Be, Fr, Po and Sp). BayeScan assumes that divergence at each locus between populations is the result of population specific divergence from an ancestral population as well as a locus specific effect. The prior odds of the neutral model was set to 10. Twenty pilot runs, 5,000 iterations each, were set to optimize proposal distributions and final runs were performed for 50,000 iterations, outputting every tenth iteration, and a burn-in of 50,000 iterations. Detection of outlier loci is particularly vulnerable to false positives [76]. To account for this, we applied a false discovery rate (FDR) correction of 0.05, meaning that the expected proportion of false positives is 5% [34].

To test for the presence of SNPs whose alleles are directionally selected in the two habitats across all populations, we used the approach implemented in BayEnv2 [35,72]. This method identifies SNPs whose allele frequencies are strongly correlated with an environmental variable given the overall covariance in allele frequencies among populations. The covariance in allele frequencies, which represents the null model against which the effect, *β*, of an environmental variable on the allele frequencies of each SNP is tested, was estimated based on all SNPs present in at least 80% of the individuals. This covariance matrix was strongly correlated with the *F*_*ST*_ matrix (Mantel-test: *r*_*S*_ = 0.87), indicating that it accurately reflects the genetic structuring of the populations. For each SNP, the posterior probability of a null model assuming no effect of the environment (*β* = 0) is compared against the alternative model which includes the effect of the environmental variable. As environmental variable, we assigned tidal habitats (BeS, FrS, PoS, SpS and UkS) the value -1 and seasonal inundated habitats (BeL, FrL, PoL, SpL and MeL) the value 1. The degree of support that variation at a SNP covaries with the habitat wherein the population was sampled is then given by the Bayes Factor (BF), the ratio of the posterior probabilities of the alternative versus the null model. For both the estimation of the covariance structure and the environmental effect, a total of 100,000 iterations was specified.

### Reconstructing the evolutionary history of outlier loci

To gain insight into the evolutionary history of the alleles differentiating the two ecotypes, we reconstructed haplotypes of the 1200 bp long paired RAD-tag loci for each individual. Sites with a read depth lower than 10 or a genotype quality lower than 20 were treated as missing. Haplotypes could be reconstructed for 627 paired RAD-tags with on average 671 bp genotyped in at least 75% of the individuals. We constructed split networks with the NeighbourNet algorithm using SplitsTree4 [77] for all RAD-tags that contained an outlier SNP with a Bayes Factor (BF) support level larger than 3 based on the BayEnv2 analysis. Haplotypes were subsequently split in two groups according to base composition at the outlier SNP with the highest support and visualized on the networks.

We further calculated for all RAD-tags the following haplotype statistics with the EggLib v2.1.10 Python library [78]: haplotype based *F*_*ST*_ [79], average pairwise difference (*d*_*XY*_) between both ecotypes, total nucleotide diversity (*π*_tot_), nucleotide diversity within the long- and short-winged ecotype (*π*_L_ and *π*_S_, respectively) and Tajima’s *D*. Comparison of measures of *d*_*XY*_ (≈ 2*μ*t + θ_Anc_) and *π* (≈ 4*Nμ*) between RAD-tags depend, besides the average coalescence time between haplotypes, also on the mutation rate (*μ*) of the RAD-tag. As we are primarily interested in comparing values of these statistics among RAD-tags independent of their mutation rate, we normalized these values by the average number of nucleotide differences between *P*. *chalceus* and the outgroup species *P*. *littoralis* [80]. More specifically, we first calculated *d*_*XY*_ between haplotypes of *P*. *chalceus* and *P*. *littoralis* (*d*_*xy*, littoralis_), and divided both *π*_tot_ and *d*_*XY*_ by this value.

We estimated the divergence time between haplotypes selected in short- and long-winged populations with BEAST 1.7.1 [38]. The analysis was restricted to outlier RAD-tags (15 in total) that are also present in the outgroup species *P*. *littoralis* and that contained on average at least 10 segregating sites among the sequences of *P*. *chalceus*. This latter criterium was implemented to ensure a sufficiently high substitution rate for reliable time calibration. The tree was calibrated using the divergence from *P*. *littoralis*, estimated at 0.62 ± 0.06MY, as calibration point [36]. We assumed a GTR substitution model, a strict clock model and standard coalescent tree prior. Analyses were run by default for 10 million generations of which the first 2 million generations were treated as burn-in and discarded for the calculation of posterior probability estimates.

### Data accessibility

Raw sequencing reads are available in the NCBI Short Read Archive under BioProject PRJNA381601. The genome assembly, ordered using the linkage map, is available under accession NEEE00000000. Reads of genome assembly: SAMN06684244-SAMN06684249; RAD-seq data of *Pogonus chalceus*: SAMN06691389-SAMN06691532; RAD-seq data of *Pogonus littoralis*: SAMN06691533-SAMN06691534; RAD-seq data for linkage map construction: SAMN06806679- SAMN06806758. The genotype VCF file, population genetic statistics and *δ*a*δ*i, BayEnv and BayeScan results can be found on dryad: doi:10.5061/dryad.77r93d5.

## Supporting information

S1 Supporting Methods Genome assembly, linkage map and outlier loci. (DOCX)

S1 Supporting Results *P. chalceus* wing-size dynamics in a newly colonized isolated tidal marsh. (DOCX)

S1 Table Average nucleotide diversity (π ± SD) across RAD-tags per population and average *F*_ST_-values across SNPs (1 SNP per paired RAD-tag) for all pairwise population comparisons. Below diagonal: *F*_ST_ based on all RAD-tags. Above diagonal: *F*_ST_ based on the ‘neutral’ set of RAD-tags.
(PDF)

S2 Table Demographic parameters estimated with *δ*a*δ*i. The demographic inference was based on the joint allele frequency spectrum (JAFS) assuming a secondary contact model with heterogeneous genomic divergence and background selection (SC2M_hrf) for the among ecotype comparisons and a secondary contact model with homogeneous genomic divergence and background selection (SC_hrf) for the within ecotype comparisons. Parameters indicated with an (*) are scaled to ancestral population mutation rate. *v* = relative size of the subpopulations compared to the ancestral population, *hrf* = scaling factor of the reduction in population size due to sites linked to sites under background selection, *M* = population migration rate (number of copies), *M*(I) = population migration rate (number of copies) in genomic islands, *t*_s_ = Time of the split between the ecotypes, *t*_sc_ = Time of the onset of secondary contact, *Q* = proportion of the genome linked to sites under background selection, *P* = proportion of the genome located within genomic islands, *θ* = population mutation rate, *N* = population size expressed in number of individuals, *m* = migration rate (proportion), *m*(I) = migration rate (proportion) within genomic islands. L, S and A refer to the long-winged ecotype, short-winged ecotype and ancestral population respectively.
(PDF)

S3 Table Sampling information for RAD-tag sequencing. EL = elytral length, EW = elytral width, WL = wing length, WW = wing width.
(PDF)

S4 Table *Pogonus chalceus* genome sequencing read statistics. Error corrected reads were used for SOAPdenovo2 assembly.
(PDF)

S5 Table Bacterial and viral genome assembly contamination. (PDF)

S6 Table Sample information for RAD-tag sequencing for linkage mapping. Families are separated by lines. Parents are indicated in bold. EL = elytral length, EW = elytral width, WL = wing length, WW = wing width.
(PDF)

S1 Fig Plot of the natural logarithm of the likelihood of the data for different numbers of assumed populations. *K*, as obtained from Structure v2.3. Red dots: analysis based on the complete set. Green dots: analysis based on the neutral set.
(TIF)

S2 Fig Boxplots representing the distribution of the AIC values of the different demographic models fitted with *δ*a*δ*i [33]. The three major implemented models are a strict isolation (SI), isolation with migration (IM) and a secondary contact (SC) model. Models specified with ‘-2M’ allow for a heterogeneous migration rate between the two populations to incorporate reduced migration rates in genomic islands. Models specified with ‘-hrf’ allow genomic variation in population size to incorporate selection at linked sites.
(TIF)

S3 Fig *F*_*ST*_ distribution at individual SNPs. *F*_*ST*_ distribution of all SNPs for each of the four regional ecotype comparisons and the correlation in *F*_*ST*_ among ecotype comparisons. The panels in the upper right corner show the within ecotype comparisons of populations Be and Fr. Green intensity depicts the degree of support (log_10_BF) that the alleles frequencies at each SNP is associated with the habitat type (tidal versus seasonally inundated) as determined with BayeEnv2.
(TIF)

S4 Fig Venn diagrams depicting the number of outlier loci, as identified by BayeScan v.2.1., and their proportion shared among the four different ecotype comparisons. Left Venn diagram shows the number of SNPs identified as outliers. Right Venn diagram shows the number of paired RAD-tags as outliers, wherein a paired RAD-tag containing at least one outlier SNP was considered an outlier tag. See Fig 1 for population codes.
(TIF)

S5 Fig Correlation between the level of support of outlier SNPs as identified in pairwise ecotype comparisons within regions with BayeScan v.2.1. (Qval), versus the level of support (log10BF) that allele frequencies at a SNP are correlated with habitat-type across all ten sampled populations (BayEnv2). Red dots are SNPs identified by BayeScan as outliers at a False Discovery Rate of 0.05.
(TIF)

S6 Fig Correlation between the allele frequencies at neutral versus outlier loci within chromosomes. Plots show correlation coefficient (r-squared) between allele frequencies at loci on the y-axis versus the distance (in bp) between the loci on the x-axis. Black points are r-squared values between supposedly neutral loci, whereas colored points (blue for tidal, red for seasonal populations) are r-squared values between the allele frequencies at outlier loci. The solid green line is a loess smoothed fit for the r-squared values between supposedly neutral loci. The dashed green line is a loess smoothed fit for r-squared values between the allele frequencies at outlier loci. To avoid spurious correlations coefficients due to nearly fixed variants, only loci with a minor allele frequency (MAF) of 0.1 were considered from the Be and Fr populations. R-squared values were calculated using the R package snpStats.
(TIF)

S7 Fig Chronograms of outlier loci indicating divergence time and phylogenetic relationship between short-wing tidal (blue) and long-wing seasonal (red) selected alleles in *P. chalceus*. *P*. *littoralis* (white triangle, lower clade) was used as an outgroup species. Error bars at the nodes depict the 95% CI of the node heights. Distance between vertical scale bars is 50 Kya.
(TIF)

S8 Fig Distribution of the number of markers across the 17 largest linkage groups using LOD scores ranging from 3 to 10. (TIF)

S9 Fig Linkage map of *Pogonus chalceus*. Marker density at each position is color coded with darker positions containing more markers. Markers on LG_10 are significantly sex-linked.
(TIF)
