# Supplementary material for: Evolution at two time frames: Polymorphisms from an ancient singular divergence event fuel contemporary parallel evolution
Source: PLoS Genet. 2018 Nov 13;14(11):e1007796. doi: 10.1371/journal.pgen.1007796 (PMC6258555; doi:10.1371/journal.pgen.1007796)
Supplement: S1 Supporting Methods — (DOCX) [file pgen.1007796.s001.docx]

# **Supporting methods**

# 1. Genome assembly and linkage map

To anchor our population genomic data along chromosomes, we assembled a draft genome and ordered scaffolds using a linkage map. We assembled 520 Mb of the estimated genome size of 530 Mb. After excluding contigs smaller than 500 bp, we retained 282.4 Mb of the genome sequence that was distributed over 29,219 scaffolds with an average size of 9,666 bp and an N50 of 66,457bp. The largest scaffold measured 1,116,948 bp. Mapping of the eukaryotic genes (CEG’s) to the assembled *P. chalceus* genome using the CEGMA pipeline [2], identified 230 full length and 9 partial CEGs out of the conserved set of 248 CEGs.

Linkage map reconstruction was performed with LepMAP2[3]. Posterior genotype probabilities of each individual were obtained from the SAMtools[4] mpileup output file by using the LepMAP2 associated scripts pileupParser2.awk and pileup2posterior.awk (https://sourceforge.net/projects/lepmap2/files/). At that stage, genotypes were quality filtered and only positions with a minimum read depth of 10 in at least 70 (87.5%) individuals were retained for further analysis. Genotypes of the parents, including X-linked markers, were called with a LOD score of 2 (i.e. difference between called and second most likely genotype combination) with the module ParentCall in LepMAP2. Markers showing a significant deviation from Mendelian segregation (P < 0.01) were excluded with the LepMAP2 Filter module. This resulted in a final set of 12,519 markers, of which 6,656, 5,862 and 8,392 were informative maternally, paternally or in either one of the two parents, respectively.

Markers were assigned to linkage groups (LG’s) using LOD scores ranging from 3-10 with the LepMAP2 SeperateChromosomes module. Graphical inspection of the number of markers against marker number included in each LG revealed that for LOD scores below 5, virtually all markers (> 99%) were assigned to either one single linkage group or as single markers (Figure S8). Using a LOD score of 5, there were nine LG’s with more than 300 markers and a gradual decrease in marker number from LG10 onwards. Increasing the LOD to a value of 6 resulted in a pattern with nine LG’s with more than 300 markers, two with 53 and 28 markers, respectively, and a residual large set of LG’s all with 8 or fewer markers. Increasing the LOD score to higher values resulted in a consistent pattern, wherein only the number of markers within each LG decreased. Considering the 10 + XY karyotype of *P. chalceus* [5], we assumed the largest 11 LG’s to represent the 11 chromosomes and used a final LOD score of 6 to maximize the number of included markers (*n* = 4057).

The LepMAP2 OrderMarkers module was then used for ordering the markers within LG’s while setting the prior male recombination rate equal to zero. The absence of recombination in males, due to male achiasmatic meiosis, was first verified by allowing recombination in the male parent, which confirmed that male recombination was not substantially higher than zero. We first included all markers belonging to a particular linkage group and used six iterations to estimate their order. Based on this first ordering, LepMAP2 returned a genotype error estimate for each marker. These markers may inflate the mapping distance substantially and markers with an error estimate larger than 0.1 were iteratively removed until none of the error estimates exceeded 0.1.

The X chromosome was identified by identifying markers that show a significant association (LOD > 2) with phenotypic sex (ParentCall module in LepMap2). All sex-linked markers were situated on the same linkage group LG10. Genomic scaffolds were subsequently assigned into LG’s and ordered according to the median map positions of the markers on each scaffold.

The final linkage map was based on 3,929 SNPs, distributed over these largest 11 linkage groups. These markers resulted in 460 positions on the linkage map and covered a total map length of 1897.74 cM (average length/LG = 172.52 cM) and mapped to 1,007 different scaffolds with a total length of 105 Mb (37.2% of the total draft genome assembly length) (Figure S9).

# 2. Outlier loci

We identified a total of 512 (3.2%) SNPs, situated on 109 (15%) paired RAD-tags, that showed stronger differentiation as expected by chance in at least one of the ecotype comparisons (BayeScan v2.1[6] with false discovery rate = 0.05). The number of SNPs showing those elevated levels of differentiation varied from 154 SNPs (2.0%) in Po to 351 SNPs (4.6%) in Fr and were located on 36 (6.7%; Po) to 86 (15.7%; Sp) paired RAD-tags. Although only 50 of these outlier SNPs and 17 of the paired RAD-tags containing an outlier were shared among all four population comparisons, the proportion of outlier SNPs (RAD-tags) that was unique in only one population pair was relatively low and varied from 8.6% (16.2%) in Be to 28.4% (29.9%) in Sp (Figure S2).

A total of 75 (0.48%) SNPs, distributed over 32 (6.3%) paired RAD-tags, were also identified as being significantly associated with the ecotypic divergence across all investigated populations (BayEnv2; log_10_BF = 4; Günther and Coop 2013). On average, 75% of these SNPs were also identified in the population pair comparisons described above (BayeScan v2.1). Despite this general agreement in SNPs that were consistently identified by both approaches, a few SNPs that were strongly supported to be associated with ecological divergence across the entire range were not significantly differentiated within some regional ecotype comparisons. Conversely, significant outliers at the regional level were often not supported to be outliers across the entire range and, therefore, are likely population specific (Figure S3).
