# Supplementary material for: Evolution at two time frames: Polymorphisms from an ancient singular divergence event fuel contemporary parallel evolution
Source: PLoS Genet. 2018 Nov 13;14(11):e1007796. doi: 10.1371/journal.pgen.1007796 (PMC6258555; doi:10.1371/journal.pgen.1007796)
Supplement: S1 Supporting Results — (DOCX) [file pgen.1007796.s002.docx]

**Supporting results**

# P. chalceus wing-size dynamics in a newly colonized isolated tidal marsh

We investigated the dynamics of wing size within the “Baai van Heist”, which constitutes a tidal marsh that developed within the last two decades after the completion of the harbor of Zeebrugge in 1985. Sand was deposited naturally due to the construction of a breakwater and resulted in a sandy bay of 50 ha around 1990. From 2000 onwards, vegetation started to develop along the creeks (mainly *Salicornia* sp.) and ultimately resulted in a well-developed tidal marsh from 2005 onwards (Figure 1).

The salt-marsh is inundated on an almost daily basis, and therefore expected to favor the tidal i.e. short-winged *P. chalceus* ecotype. However, as the short-winged ecotype is unable to disperse by flight, it can be expected that long-winged individuals are the first colonizing individuals of this isolated marsh. The distance to the nearest known long-winged population (Lissewege) is 4 km and 8 km from the closest short-winged population (Zwin).

The first individuals of *P. chalceus* were detected and sampled in September 2002 [1] and showed wing sizes that were not significantly different from those of the nearest long-winged population sampled at the seasonally inundated salt marsh Lissewege (Figure 2, *F* = 0.94, *P*  = 0.33). Beetles were sampled for a second time in June 2018 and wing size and elytral length were measured again using the same methodology described in [1]. Within this time span of 16 years, we observed a significant reduction in wing size (*F* = 21.8, *P* < 0.0001) and elytral length (*F* = 8.8, *P* = 0.004). This reduction in wing size persisted under a model wherein elytral length was used as a covariate (population effect: *F* = 53.2; *P* < 0.0001), showing that the reduction in wing size is partly independent of the reduction in body size.


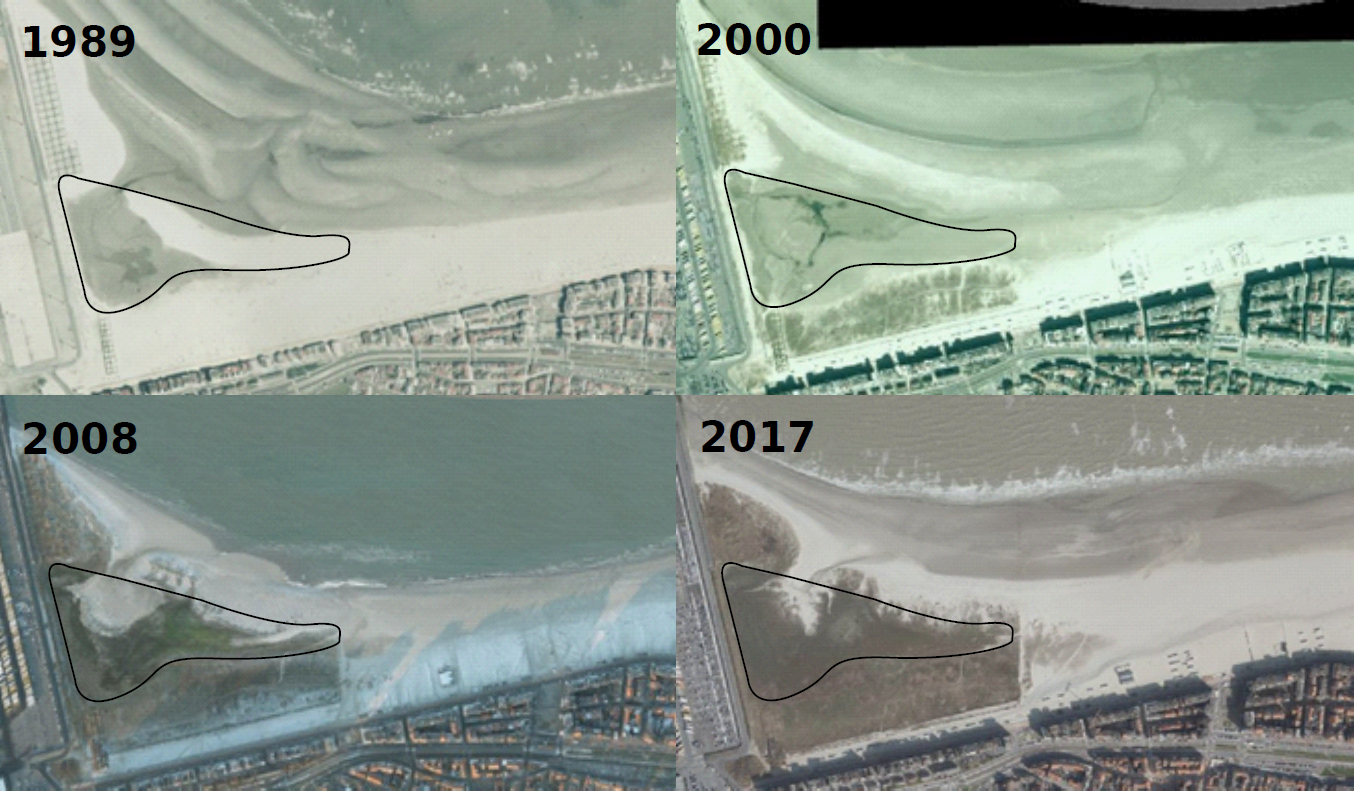


Figure 1. Salt marsh development of the “Baai van Heist” between 1989 and 2017. The black outline shows the extent of the current area of the tidal marsh ecosystem. Source pictures: “Informatie Vlaanderen” accessible through https://www.geopunt.be/.


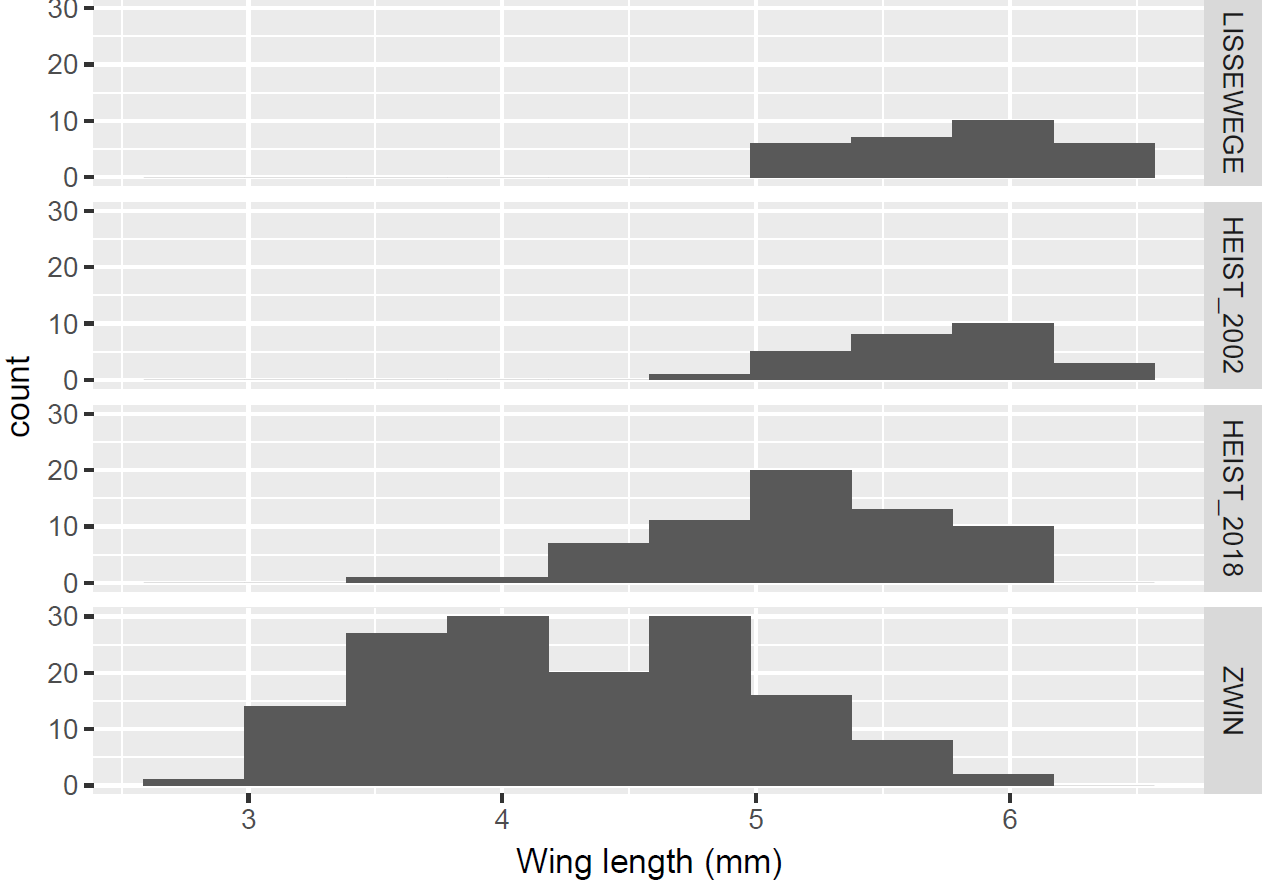

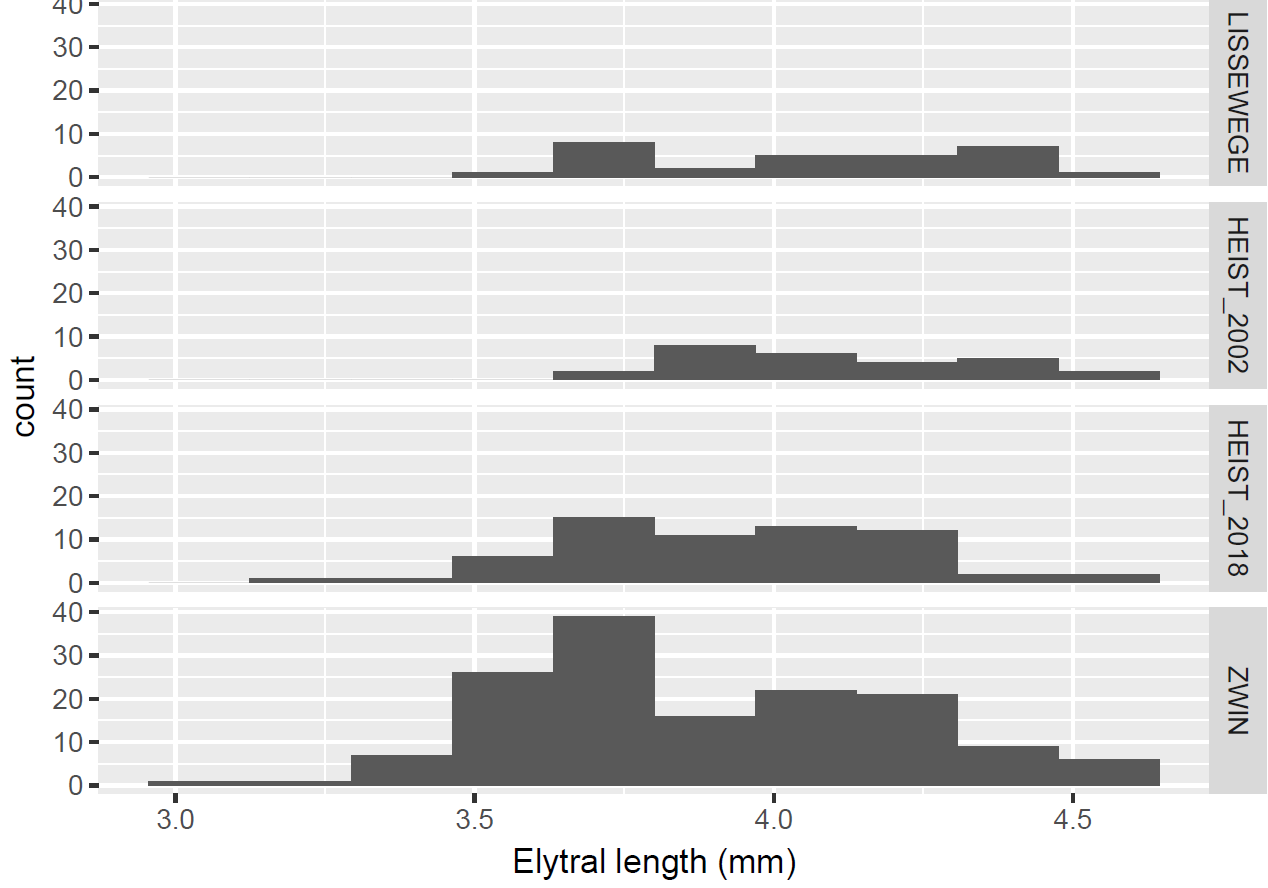


Figure 2. Distribution of wing length (left) and elytral length (right) of *P. chalceus* individuals sampled at the seasonally inundated salt marsh “Lissewege” (LISSEWEGE), the tidal inundated salt marsh “Baai van Heist” in 2002 (HEIST_2002) and 2018 (HEIST_2018) and the tidal inundated salt marsh Zwin (ZWIN).
