# Supplementary material for: Evolution at two time frames: Polymorphisms from an ancient singular divergence event fuel contemporary parallel evolution
Source: PLoS Genet. 2018 Nov 13;14(11):e1007796. doi: 10.1371/journal.pgen.1007796 (PMC6258555; doi:10.1371/journal.pgen.1007796)
Supplement: S1 Table — Below diagonal: FST based on all RAD-tags. Above diagonal: FST based on the ‘neutral’ set of RAD-tags. (PDF) [file pgen.1007796.s003.pdf]

	**π (SD)**	**Fst**									
		**BeL**	**BeS**	**FrL**	**FrS**	**PoL**	**PoS**	**SpL**	**SpS**	**MeL**	**UKS**
**BeL**	0.00408 (0.00529)	-	**0.0652**	0.1079	0.1522	0.2675	0.3041	0.2113	0.2399	0.1676	0.0793
**BeS**	0.00391 (0.00482)	**0.0918**	-	0.0970	0.0824	0.2680	0.2501	0.2072	0.1961	0.1798	0.0480
**FrL**	0.00400 (0.00504)	0.1142	0.1132	-	**0.0904**	0.2661	0.2980	0.1968	0.2262	0.1619	0.0862
**FrS**	0.00295 (0.00432)	0.2022	0.0903	**0.1358**	-	0.3015	0.2638	0.2486	0.2168	0.2327	0.0950
**PoL**	0.00360 (0.00523)	0.2497	0.2611	0.2456	0.3203	-	**0.1103**	0.1666	0.2250	0.2424	0.2400
**PoS**	0.00461 (0.00490)	0.3166	0.2411	0.3007	0.2558	**0.1552**	-	0.2650	0.2020	0.3187	0.2550
**SpL**	0.00440 (0.00564)	0.2263	0.2536	0.2220	0.3300	0.1681	0.3277	-	**0.1659**	0.1220	0.1568
**SpS**	0.00499 (0.00536)	0.2698	0.1920	0.2440	0.1999	0.2566	0.1955	**0.2700**	-	0.2392	0.1864
**MeL**	0.00532 (0.00547)	0.1778	0.2381	0.1912	0.3277	0.2401	0.3716	0.1349	0.3295	-	0.1000
**UKS**	0.00386 (0.00498)	0.0956	0.0521	0.0919	0.1218	0.2221	0.2507	0.1945	0.1968	0.1547	-
