# Supplementary material for: Evolution at two time frames: Polymorphisms from an ancient singular divergence event fuel contemporary parallel evolution
Source: PLoS Genet. 2018 Nov 13;14(11):e1007796. doi: 10.1371/journal.pgen.1007796 (PMC6258555; doi:10.1371/journal.pgen.1007796)
Supplement: S2 Table — The demographic inference was based on the joint allele frequency spectrum (JAFS) assuming a secondary contact model with heterogeneous genomic divergence and background selection (SC2M_hrf) for the among ecotype comparisons and a secondary contact model with homogeneous genomic divergence and background selection (SC_hrf) for the within ecotype comparisons. Parameters indicated with an (*) are scaled to ancestral population mutation rate. v = relative size of the subpopulations compared to the ancestral population, hrf = scaling factor of the reduction in population size due to sites linked to sites under background selection, M = population migration rate (number of copies), M(I) = population migration rate (number of copies) in genomic islands, ts = Time of the split between the ecotypes, tsc = Time of the onset of secondary contact, Q = proportion of the genome linked to sites under background selection, P = proportion of the genome located within genomic islands, θ = population mutation rate, N = population size expressed in number of individuals, m = migration rate (proportion), m(I) = migration rate (proportion) within genomic islands. L, S and A refer to the long-winged ecotype, short-winged ecotype and ancestral population respectively. (PDF) [file pgen.1007796.s004.pdf]

Among ecotypes					Within ecotypes		
**Population pair**	*Be*	*Fr*	*Po*	*Sp*	**Population pair**	*Long*	*Short*
**model**	SC2M_hrf	SC2M_hrf	SC2M_hrf	SC2M_hrf	**model**	SC_hrf	SC_hrf
***v* (L)**	[0.043-0.061]	[0.293-0.448]	[0.535-1.069]	[0.347-0.864]	***v* (Be)**	[0.065-0.113]	[0.08-0.234]
***v* (S)**	[0.199-0.28]	[0.111-0.138]	[0.007-0.014]	[0.01-0.014]	***v* (Fr)**	[0.167-0.299]	[0.162-0.286]
hrf	[0.21-0.52]	[0.06-0.34]	[0.07-0.27]	[0.03-0.44]	***hrf***	[0.14-0.51]	[0.15-0.32]
M*(L<-S)	[134.8-155.1]	[7.4-15.8]	[65.2-95.9]	[43.2-93.7]	***M*(Be<-Fr)***	[35.3-57.2]	[39.7-72.6]
M*(S<-L)	[12.1-16]	[79.4-125.5]	[381.2-656.3]	[350.3-985.2]	***M*(Fr<-Be)***	[5-6.7]	[5.1-7.4]
M*(I)(L<-S)	[24.7-29.4]	[3.9-5.7]	[0.5-4.4]	[0.4-0.8]	***M*(I)(Be<-Fr)***	-	-
M*(I)(S<-L)	[15-20]	[2.1-3.1]	[7.8-18.5]	[8.3-10.9]	***M*(I)(Fr<-Be)***	-	-
t_s*	[0.26-0.3]	[0.46-2.07]	[0.5-0.54]	[0.44-1.17]	t_s*	[0.2-0.8]	[0.38-0.98]
t_{sc}*	[0.016-0.02]	[0.063-0.135]	[0.014-0.037]	[0.011-0.023]	t_{sc}*	[0.035-0.078]	[0.037-0.072]
Q	[0.15-0.45]	[0.09-0.22]	[0.34-0.95]	[0.59-0.92]	***Q***	[0.24-0.9]	[0.1-0.53]
P	[0.24-0.37]	[0.29-0.45]	[0.17-0.47]	[0.14-0.43]	***P***	-	-
**θ (L)**	[60.9-78.7]	[362.3-428.2]	[566.2-858.9]	[435.5-707.9]	**θ (Be)**	[114.6-150.6]	[132.4-273.1]
**θ (S)**	[287.9-350.5]	[119.9-145.6]	[5-12.4]	[8.2-17.8]	**θ (Fr)**	[292-373.6]	[256.3-344.2]
**θ (A)**	[1250.9-1444.8]	[881.5-1262]	[731.2-1127.3]	[681.4-1314.3]	**θ (A)**	[1105-1752.5]	[1167.3-1810.3]
***N* (L)**	[3541-4578]	[23157-27369]	[38257-58031]	[31039-50451]	***N* (Be)**	[7420-9754]	[8423-17367]
***N* (S)**	[16750-20398]	[7661-9304]	[341-836]	[585-1268]	***N* (Fr)**	[18907-24190]	[16301-21895]
***N* (A)**	[72790-84071]	[56341-80661]	[49402-76164]	[48563-93674]	***N* (A)**	[71553-113478]	[74241-115143]
**T_s (years)**	[41043.1-45020.8]	[61347.1-233766.1]	[49754-76925.9]	[57830.5-136677]	**T_s (years)**	[34397.6-182042]	[56994.7-147984.7]
**T_{sc} (years)**	[1296.6-1786.1]	[7773.7-63327.3]	[2099.9-3643.9]	[1381.4-5330.8]	**T_{sc} (years)**	[2824-26616.7]	[4227.6-21322.1]
***m*(L<-S) (x1000)**	[0.82-1]	[0.07-0.13]	[0.51-0.88]	[0.23-0.96]	***m*(Be<-Fr) (x1000)**	[0.2-0.35]	[0.26-0.36]
***m*(S<-L) (x1000)**	[0.08-0.1]	[0.7-1.03]	[2.66-5.24]	[1.87-10.14]	***m*(Fr<-Be) (x1000)**	[0.02-0.04]	[0.03-0.04]
***m*(I)(L<-S) (x1000)**	[0.15-0.18]	[0.03-0.04]	[0-0.04]	[0-0.01]	***m*(I)(Be<-Fr) (x1000)**	-	-
***m*(I)(S<-L) (x1000)**	[0.09-0.13]	[0.02-0.03]	[0.05-0.19]	[0.05-0.11]	***m*(I)(Fr<-Be) (x1000)**	-	-
***M*(L<-S)**	[2.97-4.21]	[1.55-3.54]	[19.65-44.42]	[7.5-38.35]	***M*(Be<-Fr)**	[1.76-2.78]	[2.87-6.26]
***M*(S<-L)**	[1.41-1.99]	[5.4-8.65]	[1.54-3.07]	[2.37-5.93]	***M*(Fr<-Be)**	[0.44-0.83]	[0.49-0.74]
***M*(I)(L<-S)**	[0.53-0.77]	[0.81-1.18]	[0.14-2.02]	[0.09-0.26]	***M*(I)(Be<-Fr)**	-	-
***M*(I)(S<-L)**	[1.5-2.48]	[0.15-0.22]	[0.03-0.08]	[0.06-0.07]	***M*(I)(Fr<-Be)**	-	-
