# Supplementary material for: Evolution at two time frames: Polymorphisms from an ancient singular divergence event fuel contemporary parallel evolution
Source: PLoS Genet. 2018 Nov 13;14(11):e1007796. doi: 10.1371/journal.pgen.1007796 (PMC6258555; doi:10.1371/journal.pgen.1007796)
Supplement: S3 Table — EL = elytral length, EW = elytral width, WL = wing length, WW = wing width. (PDF) [file pgen.1007796.s005.pdf]

Sample ID	Taxon	Locality	Sex	Habitat	EL	EW	WL	WW	Accession
GC3b_071	*Pogonus chalceus*	France: Guérande	M	tidal	3.465	1.250	2.880	1.425	SAMN06691389
GC3b_072	*Pogonus chalceus*	France: Guérande	M	tidal	3.402	1.175	3.000	1.225	SAMN06691390
GC3b_073	*Pogonus chalceus*	France: Guérande	M	tidal	3.339	1.150	2.840	1.375	SAMN06691391
GC3b_074	*Pogonus chalceus*	France: Guérande	M	tidal	3.465	1.200	3.000	1.450	SAMN06691392
GC3b_075	*Pogonus chalceus*	France: Guérande	M	tidal	3.150	1.075	2.640	1.250	SAMN06691393
GC3b_076	*Pogonus chalceus*	France: Guérande	M	tidal	3.497	1.125	2.960	1.425	SAMN06691394
GC3b_077	*Pogonus chalceus*	France: Guérande	M	tidal	3.276	1.125	2.720	1.250	SAMN06691395
GC3b_078	*Pogonus chalceus*	France: Guérande	M	tidal	3.402	1.238	3.000	1.375	SAMN06691396
GC3b_079	*Pogonus chalceus*	France: Guérande	M	tidal	3.528	1.175	2.880	1.375	SAMN06691397
GC3b_080	*Pogonus chalceus*	France: Guérande	M	tidal	3.276	1.150	2.600	1.400	SAMN06691398
GC3b_081	*Pogonus chalceus*	France: Guérande	M	tidal	3.339	1.100	2.880	1.350	SAMN06691399
GC3b_082	*Pogonus chalceus*	France: Guérande	M	tidal	3.465	1.150	3.000	1.350	SAMN06691400
GC3b_083	*Pogonus chalceus*	France: Guérande	M	tidal	3.213	1.075	2.600	1.250	SAMN06691401
GC3b_084	*Pogonus chalceus*	France: Guérande	M	tidal	3.339	1.150	3.000	1.425	SAMN06691402
GC3b_085	*Pogonus chalceus*	France: Guérande	M	tidal	3.402	1.175	2.880	1.450	SAMN06691403
GC3b_086	*Pogonus chalceus*	France: Guérande	M	tidal	3.024	1.000	2.840	1.050	SAMN06691404
GC3b_087	*Pogonus chalceus*	France: Guérande	M	tidal	3.528	1.175	2.800	1.300	SAMN06691405
GC3b_088	*Pogonus chalceus*	France: Guérande	M	tidal	3.591	1.275	3.080	1.350	SAMN06691406
GC3b_089	*Pogonus chalceus*	France: Guérande	M	tidal	3.402	1.150	3.120	1.300	SAMN06691407
GC3b_090	*Pogonus chalceus*	France: Guérande	M	tidal	3.339	1.175	2.520	1.300	SAMN06691408
GC3b_091	*Pogonus chalceus*	France: Guérande	M	tidal	3.497	1.200	2.920	1.425	SAMN06691409
GC3b_092	*Pogonus chalceus*	France: Guérande	M	tidal	3.623	1.175	2.920	1.400	SAMN06691410
GC3b_093	*Pogonus chalceus*	France: Guérande	M	tidal	NA	NA	NA	NA	SAMN06691411
GC3b_098	*Pogonus chalceus*	France: Guérande	M	tidal	3.402	1.125	2.680	1.225	SAMN06691412
Da_022	*Pogonus chalceus*	Belgium: Dudzele	M	seasonal	4.064	1.450	6.111	2.525	SAMN06691413
Da_029	*Pogonus chalceus*	Belgium: Dudzele	M	seasonal	3.591	1.250	5.355	2.225	SAMN06691414
Db_094	*Pogonus chalceus*	Belgium: Dudzele	M	seasonal	3.591	1.250	5.607	2.225	SAMN06691415
Db_098	*Pogonus chalceus*	Belgium: Dudzele	M	seasonal	3.591	1.325	5.355	2.225	SAMN06691416
Db_100	*Pogonus chalceus*	Belgium: Dudzele	M	seasonal	4.127	1.400	6.174	2.475	SAMN06691417
Db_101	*Pogonus chalceus*	Belgium: Dudzele	M	seasonal	3.875	1.313	5.607	2.200	SAMN06691418
Db_102	*Pogonus chalceus*	Belgium: Dudzele	M	seasonal	3.969	1.375	5.985	2.325	SAMN06691419
Db_103	*Pogonus chalceus*	Belgium: Dudzele	M	seasonal	3.591	1.300	5.292	2.225	SAMN06691420
Pc_DZ_001	*Pogonus chalceus*	Belgium: Dudzele	M	seasonal	4.032	1.350	6.174	2.500	SAMN06691421
Pc_DZ_002	*Pogonus chalceus*	Belgium: Dudzele	M	seasonal	3.717	1.250	5.733	2.200	SAMN06691422
Pc_DZ_004	*Pogonus chalceus*	Belgium: Dudzele	M	seasonal	3.969	1.350	5.733	2.225	SAMN06691423
Pc_DZ_005	*Pogonus chalceus*	Belgium: Dudzele	M	seasonal	4.001	1.325	6.143	2.400	SAMN06691424
Pc_DZ_007	*Pogonus chalceus*	Belgium: Dudzele	M	seasonal	4.095	1.375	6.111	2.450	SAMN06691425
Pc_DZ_008	*Pogonus chalceus*	Belgium: Dudzele	M	seasonal	3.843	1.350	5.733	2.325	SAMN06691426
Pc_DZ_009	*Pogonus chalceus*	Belgium: Dudzele	M	seasonal	4.064	1.400	6.269	2.650	SAMN06691427
Pc_DZ_011	*Pogonus chalceus*	Belgium: Dudzele	M	seasonal	3.780	1.325	5.922	2.250	SAMN06691428
Pc_DZ_012	*Pogonus chalceus*	Belgium: Dudzele	M	seasonal	3.812	1.350	5.796	2.375	SAMN06691429
Pc_DZ_013	*Pogonus chalceus*	Belgium: Dudzele	M	seasonal	3.875	1.350	5.922	2.375	SAMN06691430

Pc_DZ_016	*Pogonus chalceus*	Belgium: Dudzele	M	seasonal	4.127	1.425	6.363	2.563	SAMN06691431
Pc_DZ_018	*Pogonus chalceus*	Belgium: Dudzele	M	seasonal	3.969	1.375	5.733	2.275	SAMN06691432
Pc_DZ_022	*Pogonus chalceus*	Belgium: Dudzele	M	seasonal	4.032	1.300	6.174	2.400	SAMN06691433
Pc_DZ_024	*Pogonus chalceus*	Belgium: Dudzele	M	seasonal	4.032	1.450	5.670	2.250	SAMN06691434
Pc_DZ_027	*Pogonus chalceus*	Belgium: Dudzele	M	seasonal	4.127	1.425	6.426	2.575	SAMN06691435
Pc_DZ_030	*Pogonus chalceus*	Belgium: Dudzele	M	seasonal	4.127	1.425	6.426	2.550	SAMN06691436
Na_034	*Pogonus chalceus*	Belgium: Nieuwpoort	M	tidal	3.528	1.300	3.200	1.575	SAMN06691437
Nb_002	*Pogonus chalceus*	Belgium: Nieuwpoort	M	tidal	3.465	1.250	3.320	1.425	SAMN06691438
Nb_006	*Pogonus chalceus*	Belgium: Nieuwpoort	M	tidal	3.434	1.200	3.900	1.375	SAMN06691439
Nb_007	*Pogonus chalceus*	Belgium: Nieuwpoort	M	tidal	3.434	1.150	2.960	1.425	SAMN06691440
Nb_015	*Pogonus chalceus*	Belgium: Nieuwpoort	M	tidal	3.402	1.125	4.200	1.650	SAMN06691441
Nb_019	*Pogonus chalceus*	Belgium: Nieuwpoort	M	tidal	3.654	1.250	3.520	1.750	SAMN06691442
Nb_025	*Pogonus chalceus*	Belgium: Nieuwpoort	M	tidal	3.276	1.150	3.640	1.525	SAMN06691443
Nb_031	*Pogonus chalceus*	Belgium: Nieuwpoort	M	tidal	3.528	1.200	4.473	1.800	SAMN06691444
Nb_033	*Pogonus chalceus*	Belgium: Nieuwpoort	M	tidal	3.434	1.188	3.520	1.400	SAMN06691445
Nb_038	*Pogonus chalceus*	Belgium: Nieuwpoort	M	tidal	3.465	1.200	3.240	1.413	SAMN06691446
Nb_062	*Pogonus chalceus*	Belgium: Nieuwpoort	M	tidal	3.276	1.175	3.920	1.325	SAMN06691447
Nb_095	*Pogonus chalceus*	Belgium: Nieuwpoort	M	tidal	3.875	1.413	3.280	1.600	SAMN06691448
PcNP_033	*Pogonus chalceus*	Belgium: Nieuwpoort	M	tidal	3.402	1.200	3.440	1.650	SAMN06691449
PcNP_034	*Pogonus chalceus*	Belgium: Nieuwpoort	M	tidal	3.434	1.200	3.320	1.675	SAMN06691450
PcNP_035	*Pogonus chalceus*	Belgium: Nieuwpoort	M	tidal	3.213	1.100	3.000	1.425	SAMN06691451
PcNP_036	*Pogonus chalceus*	Belgium: Nieuwpoort	M	tidal	3.465	1.175	3.560	1.650	SAMN06691452
PcNP_037	*Pogonus chalceus*	Belgium: Nieuwpoort	M	tidal	3.528	1.213	3.360	1.500	SAMN06691453
PcNP_038	*Pogonus chalceus*	Belgium: Nieuwpoort	M	tidal	3.465	1.200	3.200	1.500	SAMN06691454
PcNP_040	*Pogonus chalceus*	Belgium: Nieuwpoort	M	tidal	3.213	1.125	2.940	1.400	SAMN06691455
PcNP_041	*Pogonus chalceus*	Belgium: Nieuwpoort	M	tidal	3.245	1.100	3.020	1.513	SAMN06691456
PcNP_043	*Pogonus chalceus*	Belgium: Nieuwpoort	M	tidal	3.528	1.200	3.400	1.550	SAMN06691457
PcNP_044	*Pogonus chalceus*	Belgium: Nieuwpoort	M	tidal	3.591	1.200	3.960	1.675	SAMN06691458
PcNP_045	*Pogonus chalceus*	Belgium: Nieuwpoort	M	tidal	3.528	1.188	2.960	1.500	SAMN06691459
PcNP_046	*Pogonus chalceus*	Belgium: Nieuwpoort	M	tidal	3.560	1.213	3.200	1.700	SAMN06691460
GP3_055	*Pogonus chalceus*	France: Guérande	M	seasonal	3.969	1.375	5.796	2.425	SAMN06691461
GP3_059	*Pogonus chalceus*	France: Guérande	M	seasonal	3.906	1.375	5.670	2.325	SAMN06691462
GP3_067	*Pogonus chalceus*	France: Guérande	M	seasonal	3.875	1.388	5.355	2.125	SAMN06691463
GP3_068	*Pogonus chalceus*	France: Guérande	M	seasonal	3.906	1.350	5.607	2.200	SAMN06691464
GP3_078	*Pogonus chalceus*	France: Guérande	M	seasonal	NA	NA	NA	NA	SAMN06691465
GP3_088	*Pogonus chalceus*	France: Guérande	M	seasonal	3.717	1.300	5.544	2.200	SAMN06691466
GP3b_047	*Pogonus chalceus*	France: Guérande	M	seasonal	3.843	1.300	5.166	2.075	SAMN06691467
GP3b_048	*Pogonus chalceus*	France: Guérande	M	seasonal	4.284	1.425	5.733	2.200	SAMN06691468
GP3b_055	*Pogonus chalceus*	France: Guérande	M	seasonal	4.158	1.450	5.670	2.250	SAMN06691469
GP3b_056	*Pogonus chalceus*	France: Guérande	M	seasonal	4.001	1.400	5.229	2.150	SAMN06691470
GP3b_057	*Pogonus chalceus*	France: Guérande	M	seasonal	4.032	1.375	5.733	2.275	SAMN06691471
GP3b_062	*Pogonus chalceus*	France: Guérande	M	seasonal	3.969	1.325	4.473	2.250	SAMN06691472
GP3b_063	*Pogonus chalceus*	France: Guérande	M	seasonal	3.969	1.463	4.977	1.975	SAMN06691473

GP3b_064	*Pogonus chalceus*	France: Guérande	M	seasonal	4.095	1.450	5.922	2.150	SAMN06691474
GP3b_066	*Pogonus chalceus*	France: Guérande	M	seasonal	3.938	1.350	5.481	2.100	SAMN06691475
GP3b_067	*Pogonus chalceus*	France: Guérande	M	seasonal	4.158	1.450	5.796	2.300	SAMN06691476
GP3b_068	*Pogonus chalceus*	France: Guérande	M	seasonal	3.843	1.375	5.355	2.150	SAMN06691477
GP3b_069	*Pogonus chalceus*	France: Guérande	M	seasonal	3.906	1.350	5.292	2.150	SAMN06691478
GP3b_076	*Pogonus chalceus*	France: Guérande	M	seasonal	3.906	1.35	5.481	2.25	SAMN06691479
GP3b_077	*Pogonus chalceus*	France: Guérande	M	seasonal	4.1895	1.4	5.607	2.275	SAMN06691480
GP3b_080	*Pogonus chalceus*	France: Guérande	M	seasonal	4.158	1.5	6.111	2.25	SAMN06691481
GP3b_082	*Pogonus chalceus*	France: Guérande	M	seasonal	4.158	1.425	5.481	2.425	SAMN06691482
GP3b_084	*Pogonus chalceus*	France: Guérande	M	seasonal	4.095	1.413	5.481	2.250	SAMN06691483
GP3b_085	*Pogonus chalceus*	France: Guérande	M	seasonal	4.127	1.375	6.174	2.325	SAMN06691484
PcAVE_003	*Pogonus chalceus*	Portugal: Aveiro	M	seasonal	3.812	1.250	5.670	2.350	SAMN06691485
PcAVE_004	*Pogonus chalceus*	Portugal: Aveiro	M	seasonal	3.528	1.250	5.670	2.225	SAMN06691486
PcAVE_005	*Pogonus chalceus*	Portugal: Aveiro	M	seasonal	3.623	1.275	5.670	2.250	SAMN06691487
PcAVE_006	*Pogonus chalceus*	Portugal: Aveiro	M	seasonal	3.969	1.325	6.237	2.475	SAMN06691488
PcAVE_018	*Pogonus chalceus*	Portugal: Aveiro	F	seasonal	4.064	1.400	6.237	2.500	SAMN06691489
PcAVE_019	*Pogonus chalceus*	Portugal: Aveiro	F	seasonal	4.347	1.500	6.867	2.650	SAMN06691490
PcAVE_025	*Pogonus chalceus*	Portugal: Aveiro	F	seasonal	4.158	1.400	6.395	2.475	SAMN06691491
PcAVE_026	*Pogonus chalceus*	Portugal: Aveiro	F	seasonal	4.095	1.375	6.111	2.475	SAMN06691492
PcAVE_031	*Pogonus chalceus*	Portugal: Aveiro	M	tidal	3.213	1.025	3.280	1.475	SAMN06691493
PcAVE_032	*Pogonus chalceus*	Portugal: Aveiro	M	tidal	3.528	1.150	3.200	1.675	SAMN06691494
PcAVE_033	*Pogonus chalceus*	Portugal: Aveiro	M	tidal	3.245	1.100	3.280	1.475	SAMN06691495
PcAVE_034	*Pogonus chalceus*	Portugal: Aveiro	M	tidal	3.150	1.075	3.120	1.300	SAMN06691496
PcAVE_035	*Pogonus chalceus*	Portugal: Aveiro	F	tidal	3.717	1.225	3.640	1.525	SAMN06691497
PcAVE_036	*Pogonus chalceus*	Portugal: Aveiro	F	tidal	3.938	1.275	3.640	1.775	SAMN06691498
PcAVE_037	*Pogonus chalceus*	Portugal: Aveiro	F	tidal	3.308	1.100	3.280	1.525	SAMN06691499
PcAVE_038	*Pogonus chalceus*	Portugal: Aveiro	F	tidal	3.875	1.338	3.520	1.675	SAMN06691500
Pc277	*Pogonus chalceus*	France: Camargue	M	seasonal	3.640	1.100	5.750	2.280	SAMN06691501
Pc297	*Pogonus chalceus*	France: Camargue	F	seasonal	4.040	1.220	6.167	2.400	SAMN06691502
Pc298	*Pogonus chalceus*	France: Camargue	M	seasonal	4.040	1.280	6.083	2.480	SAMN06691503
Pc299	*Pogonus chalceus*	France: Camargue	F	seasonal	4.680	1.440	7.250	2.800	SAMN06691504
Pc300	*Pogonus chalceus*	France: Camargue	M	seasonal	3.680	1.160	5.833	2.160	SAMN06691505
Pc316	*Pogonus chalceus*	France: Camargue	F	seasonal	3.840	1.200	5.917	2.080	SAMN06691506
Pc317	*Pogonus chalceus*	France: Camargue	F	seasonal	4.120	1.280	6.250	2.400	SAMN06691507
Pc318	*Pogonus chalceus*	France: Camargue	M	seasonal	3.480	1.060	5.333	1.960	SAMN06691508
Pc786	*Pogonus chalceus*	UK: SevernEstuary	F	tidal	4.120	1.300	4.167	1.800	SAMN06691509
Pc787	*Pogonus chalceus*	UK: SevernEstuary	M	tidal	NA	NA	NA	NA	SAMN06691510
Pc788	*Pogonus chalceus*	UK: SevernEstuary	M	tidal	NA	NA	NA	NA	SAMN06691511
Pc801	*Pogonus chalceus*	UK: SevernEstuary	M	tidal	NA	NA	NA	NA	SAMN06691512
Pc805	*Pogonus chalceus*	UK: SevernEstuary	M	tidal	NA	NA	NA	NA	SAMN06691513
Pc821	*Pogonus chalceus*	UK: SevernEstuary	F	tidal	NA	NA	NA	NA	SAMN06691514
Pc822	*Pogonus chalceus*	UK: SevernEstuary	M	tidal	NA	NA	NA	NA	SAMN06691515
Pc823	*Pogonus chalceus*	UK: SevernEstuary	M	tidal	NA	NA	NA	NA	SAMN06691516

Pc2311	*Pogonus chalceus*	Spain: CotoDonana	M	seasonal	NA	NA	NA	NA	SAMN06691517
Pc2314	*Pogonus chalceus*	Spain: CotoDonana	M	seasonal	4.520	1.400	7.083	2.720	SAMN06691518
Pc2316	*Pogonus chalceus*	Spain: Huelva	M	tidal	3.560	1.080	2.667	1.200	SAMN06691519
Pc2317	*Pogonus chalceus*	Spain: Huelva	F	tidal	3.920	1.220	2.917	1.440	SAMN06691520
Pc2318	*Pogonus chalceus*	Spain: Huelva	F	tidal	4.200	1.320	2.917	1.560	SAMN06691521
Pc2321	*Pogonus chalceus*	Spain: CotoDonana	F	seasonal	4.320	1.360	6.833	2.640	SAMN06691522
Pc2323	*Pogonus chalceus*	Spain: CotoDonana	F	seasonal	4.560	1.420	7.167	2.760	SAMN06691523
Pc2324	*Pogonus chalceus*	Spain: CotoDonana	M	seasonal	4.200	1.300	6.500	2.600	SAMN06691524
Pc2325	*Pogonus chalceus*	Spain: CotoDonana	F	seasonal	4.280	1.340	6.750	2.600	SAMN06691525
Pc2331	*Pogonus chalceus*	Spain: CotoDonana	F	seasonal	4.320	1.360	6.250	2.520	SAMN06691526
Pc2332	*Pogonus chalceus*	Spain: CotoDonana	F	seasonal	4.720	1.520	7.333	2.920	SAMN06691527
Pc2336	*Pogonus chalceus*	Spain: Huelva	F	tidal	4.400	1.360	3.000	1.560	SAMN06691528
Pc2337	*Pogonus chalceus*	Spain: Huelva	F	tidal	4.000	1.240	3.083	1.480	SAMN06691529
Pc2338	*Pogonus chalceus*	Spain: Huelva	F	tidal	3.920	1.200	2.583	1.240	SAMN06691530
Pc2339	*Pogonus chalceus*	Spain: Huelva	M	tidal	3.240	1.000	2.417	0.960	SAMN06691531
Pc2340	*Pogonus chalceus*	Spain: Huelva	M	tidal	3.520	1.120	2.583	1.200	SAMN06691532
pl706	*Pogonus littoralis*	Greece: Thessaloniki	F	Axion Delta	NA	NA	NA	NA	SAMN06691533
pl707	*Pogonus littoralis*	Greece: Thessaloniki	F	Axion Delta	NA	NA	NA	NA	SAMN06691534
