# Supplementary material for: Evolution at two time frames: Polymorphisms from an ancient singular divergence event fuel contemporary parallel evolution
Source: PLoS Genet. 2018 Nov 13;14(11):e1007796. doi: 10.1371/journal.pgen.1007796 (PMC6258555; doi:10.1371/journal.pgen.1007796)
Supplement: S4 Table — Error corrected reads were used for SOAPdenovo2 assembly. (PDF) [file pgen.1007796.s006.pdf]

Individual	sex	Library	Insert (bp)	GC %	Raw reads Length (bp)	Raw reads Reads (M)	Raw reads Bases (Gb)	Cleaned (and error corrected) Length (bp)	Cleaned (and error corrected) Reads (M)	Cleaned (and error corrected) Bases (Gb)	Cleaned (and error corrected) Corr. bases (Mb)	Accession
GC3b_01	m	Paired-end	200	28	101	107.24	10.83	100.04	107.06	10.71	2.18	SAMN06684244
GC3b_02	m	Paired-end	200	28	101	72.84	7.36	99.92	72.66	7.26	1.89	SAMN06684245
GC3b_03	m	Paired-end	200	28	101	53.92	5.46	99.70	53.76	5.36	1.59	SAMN06684246
GC3b_04	m	Paired-end	200	28	101	101.3	10.23	99.97	101.13	10.11	2.40	SAMN06684247
GC3b_055L10	f	Paired-end	500	28	100	79.46	7.95	99.17	79.26	7.86	2.29	SAMN06684248
GC3b_055L10	f	Paired-end	800	28	100	61.74	6.17	99.10	61.54	6.10	2.44	SAMN06684248
GC3b_055L10	f	Mate-pair	2,000	29	49	112.18	5.50	47.34	103.26	4.89	2.57	SAMN06684248
GC3b_003	f	Mate-pair	5,000	29	49	87.14	4.27	47.24	79.78	3.77	1.77	SAMN06684249
Total						675.82	57.77		658.45	56.06	17.13	
