# Supplementary material for: Evolution at two time frames: Polymorphisms from an ancient singular divergence event fuel contemporary parallel evolution
Source: PLoS Genet. 2018 Nov 13;14(11):e1007796. doi: 10.1371/journal.pgen.1007796 (PMC6258555; doi:10.1371/journal.pgen.1007796)
Supplement: S5 Table — (PDF) [file pgen.1007796.s007.pdf]

**Species**	**Number**
Rickettsia sp.	106
Wolbachia sp.	14
Staphylococcus aureus	6
Staphylococcus phage	6
Other bacterial species	6
**Total**	**137**
