# Supplementary material for: Evolution at two time frames: Polymorphisms from an ancient singular divergence event fuel contemporary parallel evolution
Source: PLoS Genet. 2018 Nov 13;14(11):e1007796. doi: 10.1371/journal.pgen.1007796 (PMC6258555; doi:10.1371/journal.pgen.1007796)
Supplement: S6 Table — Families are separated by lines. Parents are indicated in bold. EL = elytral length, EW = elytral width, WL = wing length, WW = wing width. (PDF) [file pgen.1007796.s008.pdf]

Sample ID	Taxon	Locality	Sex	Habitat	EL	EW	WL	WW	Accession
**GC3b_026L4**	*Pogonus chalceus*	France: Guérande	m	tidal	3.717	1.100	4.662	1.300	SAMN06806679
**GC3b_007L10**	*Pogonus chalceus*	France: Guérande	f	tidal	3.339	1.325	3.160	1.450	SAMN06806680
GC3b_007L10A1	*Pogonus chalceus*	France: Guérande	f	tidal	3.245	1.150	2.800	1.200	SAMN06806681
GC3b_007L10A11	*Pogonus chalceus*	France: Guérande	m	tidal	3.465	1.150	2.980	1.300	SAMN06806682
GC3b_007L10A12	*Pogonus chalceus*	France: Guérande	m	tidal	3.276	1.125	2.898	1.300	SAMN06806683
GC3b_007L10A14	*Pogonus chalceus*	France: Guérande	f	tidal	2.898	0.875	2.680	1.025	SAMN06806684
GC3b_007L10A15	*Pogonus chalceus*	France: Guérande	f	tidal	3.591	1.300	3.040	1.375	SAMN06806685
GC3b_007L10A17	*Pogonus chalceus*	France: Guérande	f	tidal	3.843	1.250	3.080	1.375	SAMN06806686
GC3b_007L10A18	*Pogonus chalceus*	France: Guérande	f	tidal	3.213	1.200	2.960	1.075	SAMN06806687
GC3b_007L10A2	*Pogonus chalceus*	France: Guérande	m	tidal	2.993	1.100	2.760	1.200	SAMN06806688
GC3b_007L10A21	*Pogonus chalceus*	France: Guérande	m	tidal	2.961	1.025	2.680	1.225	SAMN06806689
GC3b_007L10A23	*Pogonus chalceus*	France: Guérande	m	tidal	2.835	1.000	NA	NA	SAMN06806690
GC3b_007L10A24	*Pogonus chalceus*	France: Guérande	m	tidal	2.993	1.025	2.800	1.200	SAMN06806691
GC3b_007L10A26	*Pogonus chalceus*	France: Guérande	f	tidal	3.150	1.050	2.600	1.200	SAMN06806692
GC3b_007L10A27	*Pogonus chalceus*	France: Guérande	f	tidal	3.528	1.250	2.760	1.400	SAMN06806693
GC3b_007L10A28	*Pogonus chalceus*	France: Guérande	f	tidal	3.528	1.138	2.840	1.325	SAMN06806694
GC3b_007L10A30	*Pogonus chalceus*	France: Guérande	m	tidal	3.276	1.200	2.680	1.250	SAMN06806695
GC3b_007L10A31	*Pogonus chalceus*	France: Guérande	m	tidal	3.339	1.175	2.880	1.250	SAMN06806696
GC3b_007L10A32	*Pogonus chalceus*	France: Guérande	f	tidal	3.591	1.200	2.940	1.250	SAMN06806697
GC3b_007L10A33	*Pogonus chalceus*	France: Guérande	f	tidal	3.402	1.175	2.772	1.175	SAMN06806698
GC3b_007L10A34	*Pogonus chalceus*	France: Guérande	m	tidal	3.150	1.250	2.800	1.200	SAMN06806699
GC3b_007L10A4	*Pogonus chalceus*	France: Guérande	f	tidal	3.528	1.238	2.560	1.300	SAMN06806700
GC3b_007L10A5	*Pogonus chalceus*	France: Guérande	f	tidal	3.087	1.025	2.560	1.075	SAMN06806701
GC3b_007L10A6	*Pogonus chalceus*	France: Guérande	m	tidal	3.528	1.188	3.040	1.375	SAMN06806702
GC3b_007L10A9	*Pogonus chalceus*	France: Guérande	m	tidal	3.276	1.100	2.760	1.200	SAMN06806703
**GC3b_053L13**	*Pogonus chalceus*	France: Guérande	m	tidal	3.339	1.000	2.760	1.300	SAMN06806704
**GC3b_026L17**	*Pogonus chalceus*	France: Guérande	f	tidal	3.969	1.300	3.280	1.650	SAMN06806705
GC3b_026L17A1	*Pogonus chalceus*	France: Guérande	f	tidal	3.749	1.250	3.080	1.325	SAMN06806706
GC3b_026L17A10	*Pogonus chalceus*	France: Guérande	f	tidal	3.780	1.300	2.760	1.400	SAMN06806707
GC3b_026L17A11	*Pogonus chalceus*	France: Guérande	m	tidal	3.024	1.225	2.600	1.125	SAMN06806708
GC3b_026L17A14	*Pogonus chalceus*	France: Guérande	f	tidal	4.032	1.350	2.920	1.500	SAMN06806709
GC3b_026L17A15	*Pogonus chalceus*	France: Guérande	f	tidal	3.969	1.300	3.024	1.450	SAMN06806710
GC3b_026L17A17	*Pogonus chalceus*	France: Guérande	f	tidal	3.843	1.250	3.020	1.525	SAMN06806711
GC3b_026L17A18	*Pogonus chalceus*	France: Guérande	m	tidal	3.182	1.100	2.820	1.175	SAMN06806712
GC3b_026L17A21	*Pogonus chalceus*	France: Guérande	f	tidal	3.591	1.263	2.840	1.250	SAMN06806713
GC3b_026L17A23	*Pogonus chalceus*	France: Guérande	m	tidal	3.528	1.175	2.920	1.350	SAMN06806714
GC3b_026L17A25	*Pogonus chalceus*	France: Guérande	f	tidal	3.465	1.200	2.840	1.200	SAMN06806715
GC3b_026L17A5	*Pogonus chalceus*	France: Guérande	f	tidal	3.843	1.313	3.000	1.425	SAMN06806716
GC3b_026L17A6	*Pogonus chalceus*	France: Guérande	m	tidal	3.591	1.200	3.000	1.375	SAMN06806717
GC3b_026L17A7	*Pogonus chalceus*	France: Guérande	m	tidal	3.087	1.050	2.560	1.225	SAMN06806718
GC3b_026L17A9	*Pogonus chalceus*	France: Guérande	m	tidal	3.497	1.125	2.940	1.325	SAMN06806719

**GC3b_026L2**	*Pogonus chalceus*	France: Guérande	m	tidal	3.591	1.125	3.240	1.450	SAMN06806720
**GP3b_005L1**	*Pogonus chalceus*	France: Guérande	f	tidal	NA	NA	NA	NA	SAMN06806721
GP3b_005L1A11	*Pogonus chalceus*	France: Guérande	f	tidal	4.064	1.325	4.158	1.900	SAMN06806722
GP3b_005L1A13	*Pogonus chalceus*	France: Guérande	f	tidal	3.812	1.275	3.920	1.850	SAMN06806723
GP3b_005L1A15	*Pogonus chalceus*	France: Guérande	m	tidal	3.843	1.350	2.640	1.950	SAMN06806724
GP3b_005L1A16	*Pogonus chalceus*	France: Guérande	m	tidal	3.560	1.175	5.859	1.800	SAMN06806725
GP3b_005L1A18	*Pogonus chalceus*	France: Guérande	m	tidal	3.717	1.300	3.000	1.725	SAMN06806726
GP3b_005L1A19	*Pogonus chalceus*	France: Guérande	f	tidal	4.221	1.475	3.880	2.100	SAMN06806727
GP3b_005L1A2	*Pogonus chalceus*	France: Guérande	f	tidal	3.528	1.188	4.347	1.675	SAMN06806728
GP3b_005L1A20	*Pogonus chalceus*	France: Guérande	f	tidal	4.379	1.500	4.095	2.125	SAMN06806729
GP3b_005L1A21	*Pogonus chalceus*	France: Guérande	m	tidal	3.339	1.150	4.284	1.725	SAMN06806730
GP3b_005L1A23	*Pogonus chalceus*	France: Guérande	f	tidal	4.095	1.400	4.347	2.025	SAMN06806731
GP3b_005L1A24	*Pogonus chalceus*	France: Guérande	m	tidal	3.780	1.300	6.174	1.900	SAMN06806732
GP3b_005L1A26	*Pogonus chalceus*	France: Guérande	f	tidal	4.316	1.450	4.410	2.225	SAMN06806733
GP3b_005L1A28	*Pogonus chalceus*	France: Guérande	f	tidal	3.969	1.375	3.920	1.950	SAMN06806734
GP3b_005L1A29	*Pogonus chalceus*	France: Guérande	f	tidal	4.032	1.325	4.284	2.025	SAMN06806735
GP3b_005L1A3	*Pogonus chalceus*	France: Guérande	f	tidal	4.158	1.375	3.920	2.000	SAMN06806736
GP3b_005L1A30	*Pogonus chalceus*	France: Guérande	f	tidal	3.780	1.450	4.788	1.900	SAMN06806737
GP3b_005L1A32	*Pogonus chalceus*	France: Guérande	f	tidal	3.434	1.225	4.095	1.600	SAMN06806738
GP3b_005L1A33	*Pogonus chalceus*	France: Guérande	f	tidal	4.221	1.425	3.560	2.075	SAMN06806739
GP3b_005L1A34	*Pogonus chalceus*	France: Guérande	f	tidal	4.001	1.275	3.680	1.900	SAMN06806740
GP3b_005L1A35	*Pogonus chalceus*	France: Guérande	f	tidal	3.843	1.325	4.253	1.950	SAMN06806741
GP3b_005L1A6	*Pogonus chalceus*	France: Guérande	m	tidal	3.591	1.200	6.300	1.825	SAMN06806742
GP3b_005L1A7	*Pogonus chalceus*	France: Guérande	f	tidal	4.032	1.375	4.040	2.025	SAMN06806743
GP3b_005L1A8	*Pogonus chalceus*	France: Guérande	m	tidal	3.465	1.175	4.158	1.825	SAMN06806744
**GP3b_005L6**	*Pogonus chalceus*	France: Guérande	m	tidal	3.906	1.225	5.481	1.925	SAMN06806745
**GP3b_031L2**	*Pogonus chalceus*	France: Guérande	f	tidal	3.969	1.325	4.221	2.000	SAMN06806746
GP3b_031L2A1	*Pogonus chalceus*	France: Guérande	m	tidal	3.623	1.225	3.760	1.750	SAMN06806747
GP3b_031L2A10	*Pogonus chalceus*	France: Guérande	m	tidal	3.434	1.175	2.720	1.650	SAMN06806748
GP3b_031L2A11	*Pogonus chalceus*	France: Guérande	f	tidal	4.064	1.375	5.418	2.000	SAMN06806749
GP3b_031L2A12	*Pogonus chalceus*	France: Guérande	f	tidal	4.064	1.450	5.607	2.100	SAMN06806750
GP3b_031L2A18	*Pogonus chalceus*	France: Guérande	m	tidal	3.717	1.300	5.040	2.000	SAMN06806751
GP3b_031L2A2	*Pogonus chalceus*	France: Guérande	f	tidal	4.158	1.500	3.720	1.950	SAMN06806752
GP3b_031L2A23	*Pogonus chalceus*	France: Guérande	m	tidal	3.339	1.225	3.920	1.800	SAMN06806753
GP3b_031L2A3	*Pogonus chalceus*	France: Guérande	f	tidal	4.158	1.375	4.020	2.175	SAMN06806754
GP3b_031L2A6	*Pogonus chalceus*	France: Guérande	f	tidal	4.095	1.425	5.229	1.850	SAMN06806755
GP3b_031L2A7	*Pogonus chalceus*	France: Guérande	m	tidal	3.938	1.350	4.662	2.050	SAMN06806756
GP3b_031L2A8	*Pogonus chalceus*	France: Guérande	f	tidal	4.221	1.425	5.607	2.275	SAMN06806757
GP3b_031L2A9	*Pogonus chalceus*	France: Guérande	m	tidal	3.654	1.250	4.095	2.025	SAMN06806758
