# Supplementary figures and images for: Evolution at two time frames: Polymorphisms from an ancient singular divergence event fuel contemporary parallel evolution
Source: PLoS Genet. 2018 Nov 13;14(11):e1007796. doi: 10.1371/journal.pgen.1007796 (PMC6258555; doi:10.1371/journal.pgen.1007796)

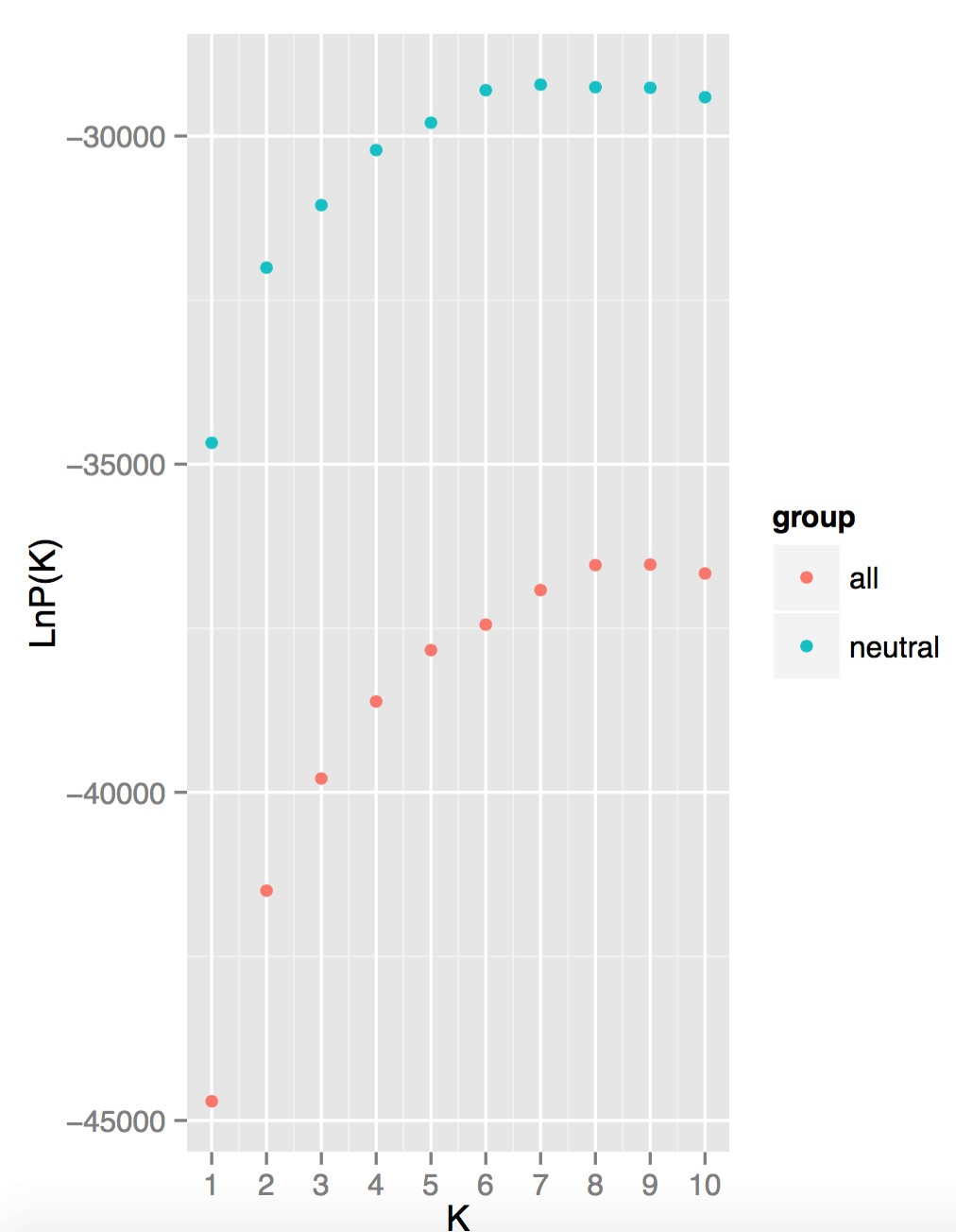

Supplement: S1 Fig — K, as obtained from Structure v2.3. Red dots: analysis based on the complete set. Green dots: analysis based on the neutral set. (TIF) [file pgen.1007796.s009.tif]

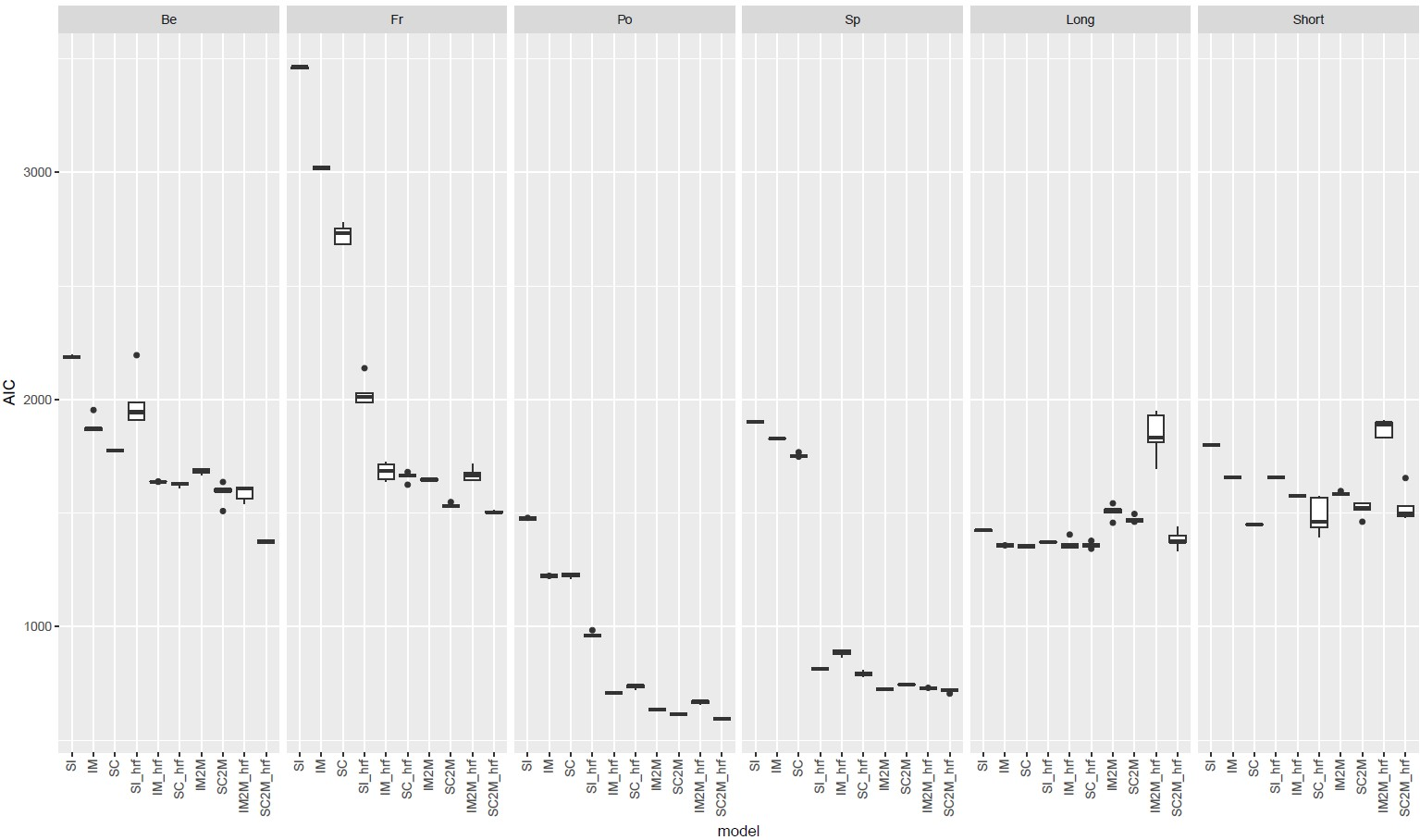

Supplement: S2 Fig — The three major implemented models are a strict isolation (SI), isolation with migration (IM) and a secondary contact (SC) model. Models specified with ‘-2M’ allow for a heterogeneous migration rate between the two populations to incorporate reduced migration rates in genomic islands. Models specified with ‘-hrf’ allow genomic variation in population size to incorporate selection at linked sites. (TIF) [file pgen.1007796.s010.tif]

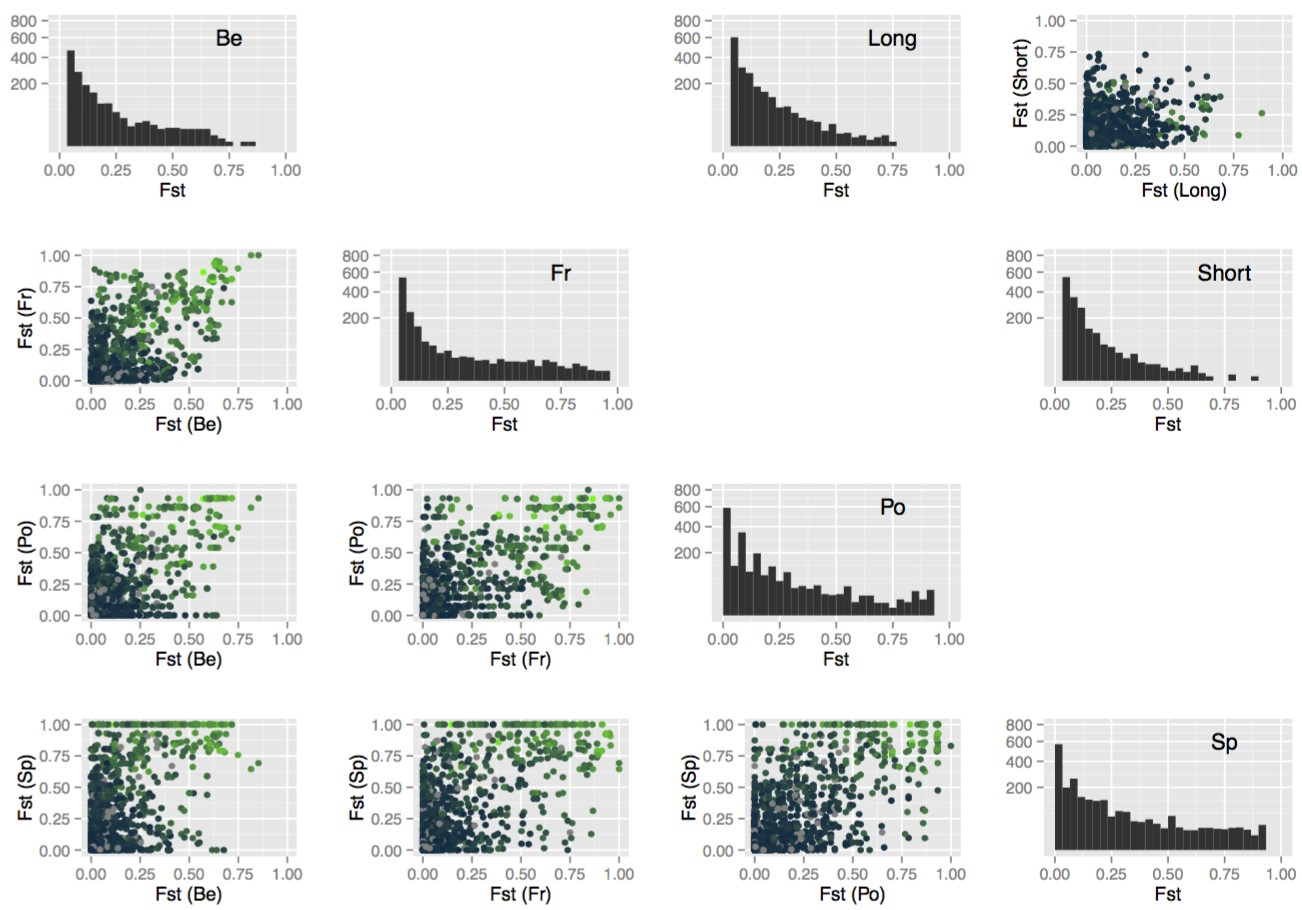

Supplement: S3 Fig — FST distribution of all SNPs for each of the four regional ecotype comparisons and the correlation in FST among ecotype comparisons. The panels in the upper right corner show the within ecotype comparisons of populations Be and Fr. Green intensity depicts the degree of support (log10BF) that the alleles frequencies at each SNP is associated with the habitat type (tidal versus seasonally inundated) as determined with BayeEnv2. (TIF) [file pgen.1007796.s011.tif]

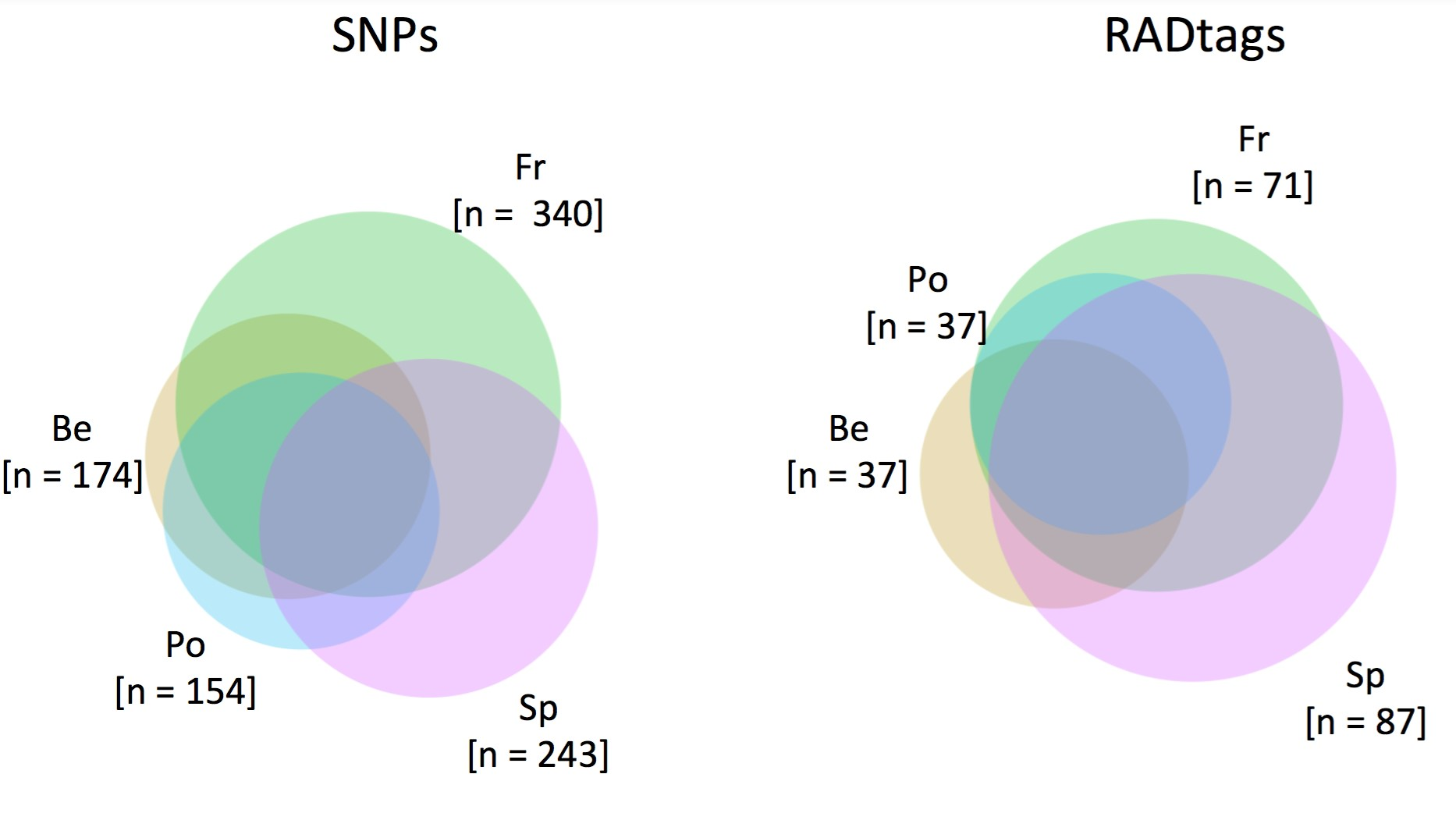

Supplement: S4 Fig — Left Venn diagram shows the number of SNPs identified as outliers. Right Venn diagram shows the number of paired RAD-tags as outliers, wherein a paired RAD-tag containing at least one outlier SNP was considered an outlier tag. See Fig 1 for population codes. (TIF) [file pgen.1007796.s012.tif]

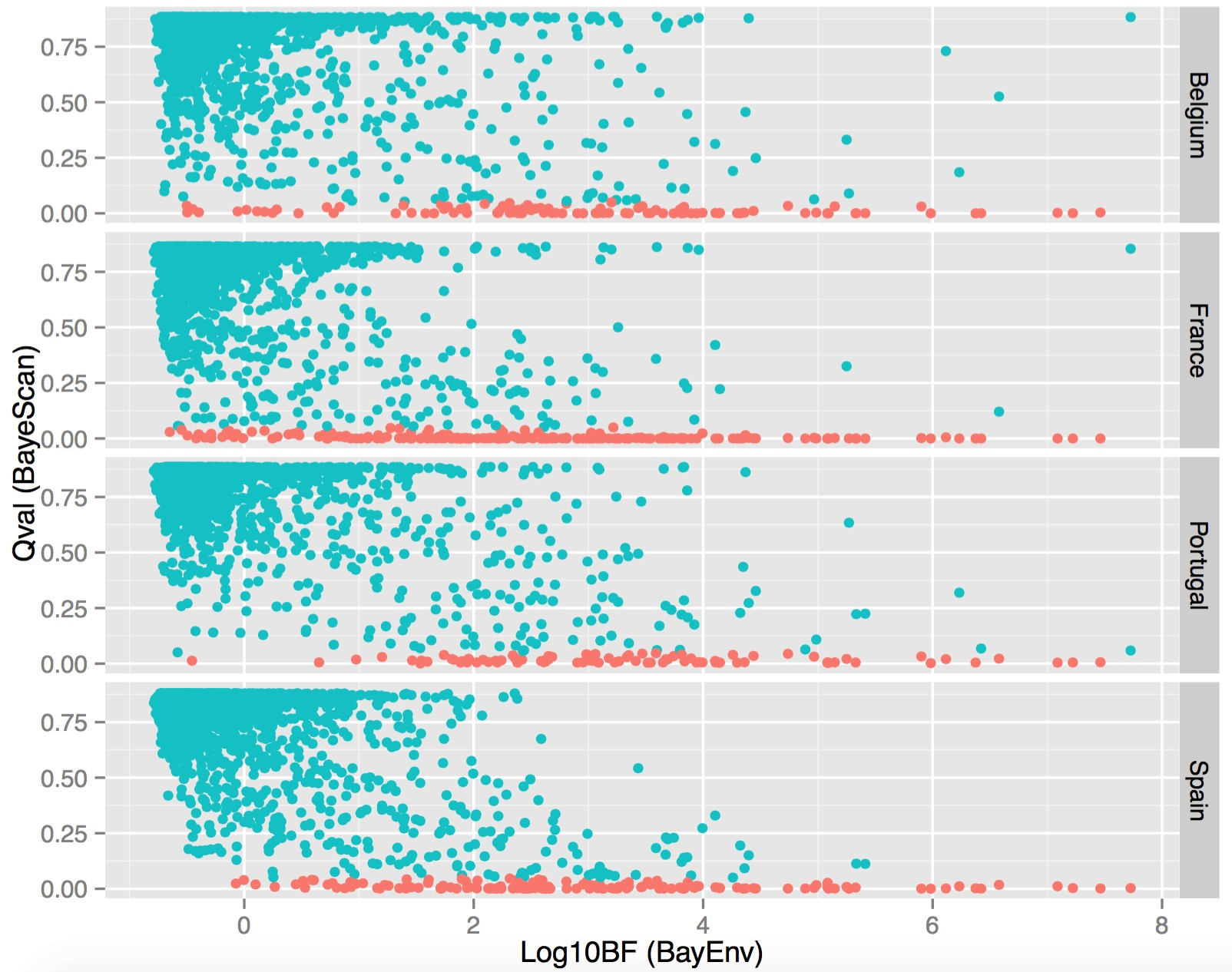

Supplement: S5 Fig — (Qval), versus the level of support (log10BF) that allele frequencies at a SNP are correlated with habitat-type across all ten sampled populations (BayEnv2). Red dots are SNPs identified by BayeScan as outliers at a False Discovery Rate of 0.05. (TIF) [file pgen.1007796.s013.tif]

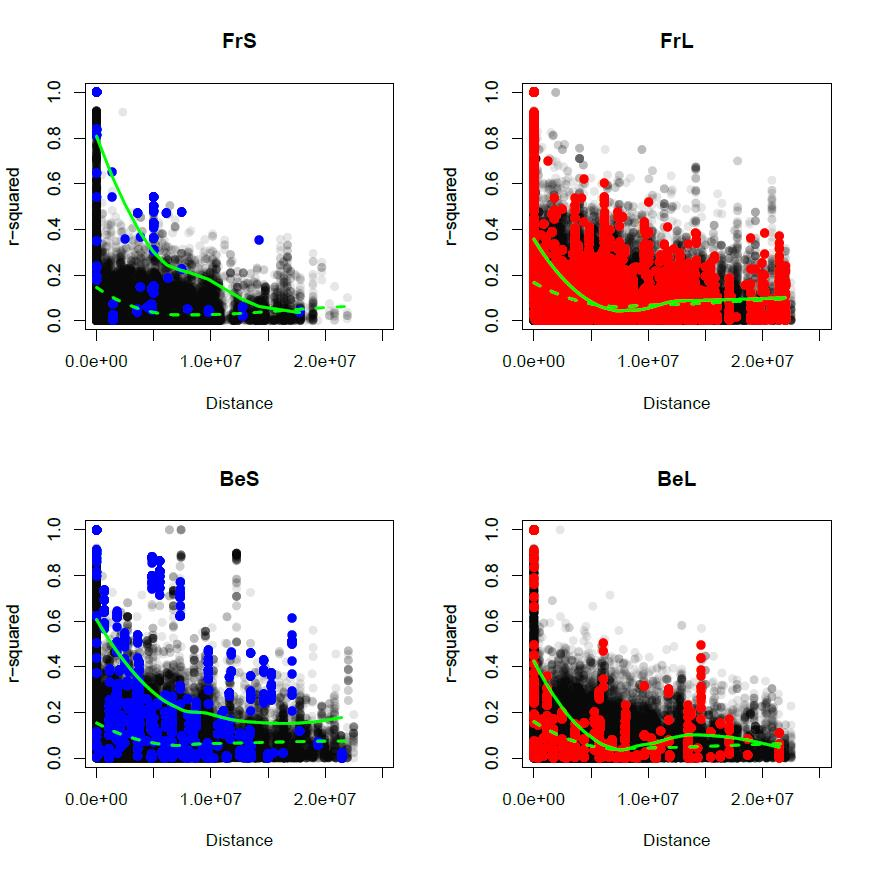

Supplement: S6 Fig — Plots show correlation coefficient (r-squared) between allele frequencies at loci on the y-axis versus the distance (in bp) between the loci on the x-axis. Black points are r-squared values between supposedly neutral loci, whereas colored points (blue for tidal, red for seasonal populations) are r-squared values between the allele frequencies at outlier loci. The solid green line is a loess smoothed fit for the r-squared values between supposedly neutral loci. The dashed green line is a loess smoothed fit for r-squared values between the allele frequencies at outlier loci. To avoid spurious correlations coefficients due to nearly fixed variants, only loci with a minor allele frequency (MAF) of 0.1 were considered from the Be and Fr populations. R-squared values were calculated using the R package snpStats. (TIF) [file pgen.1007796.s014.tif]

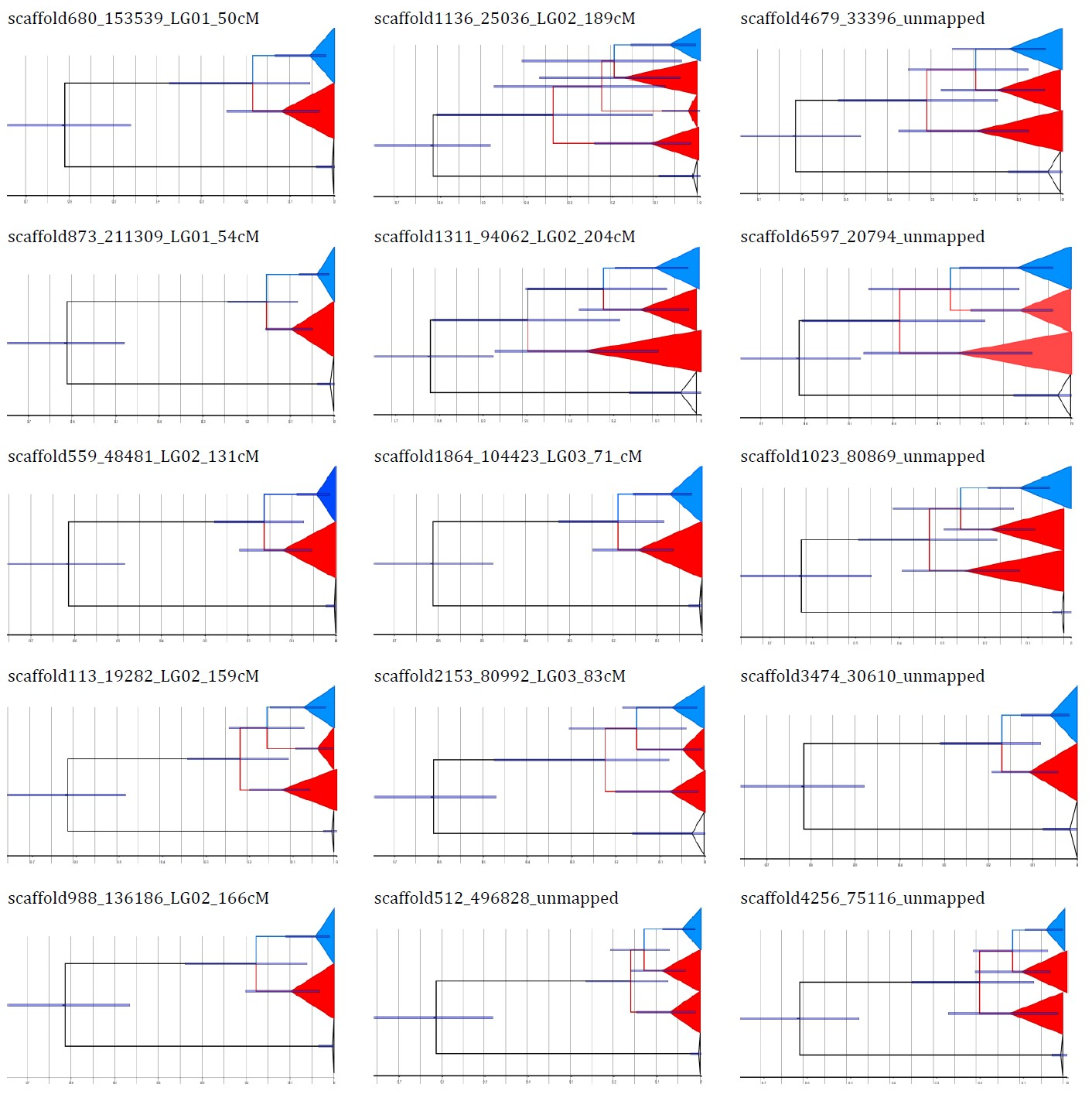

Supplement: S7 Fig — P. littoralis (white triangle, lower clade) was used as an outgroup species. Error bars at the nodes depict the 95% CI of the node heights. Distance between vertical scale bars is 50 Kya. (TIF) [file pgen.1007796.s015.tif]

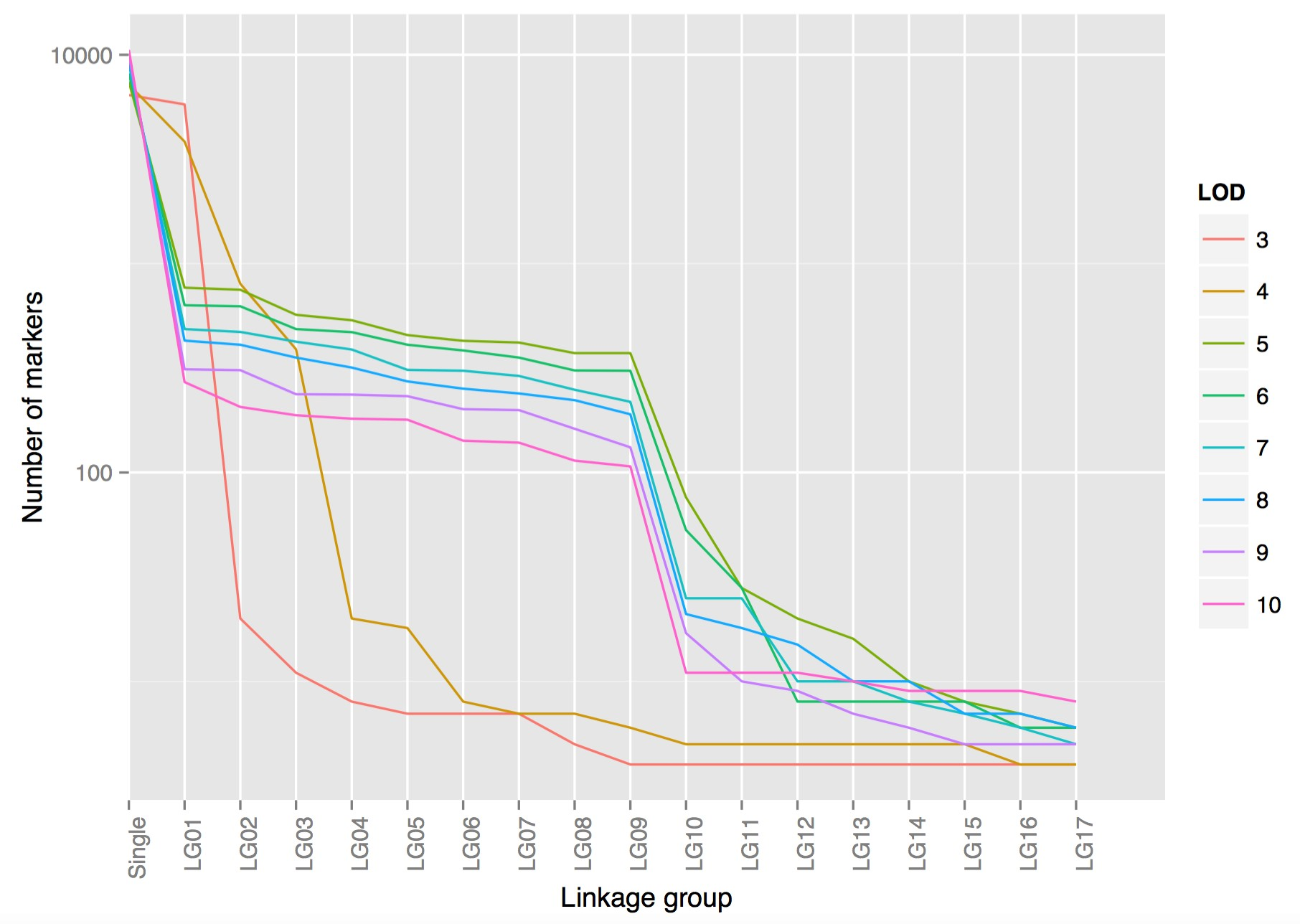

Supplement: S8 Fig — (TIF) [file pgen.1007796.s016.tif]

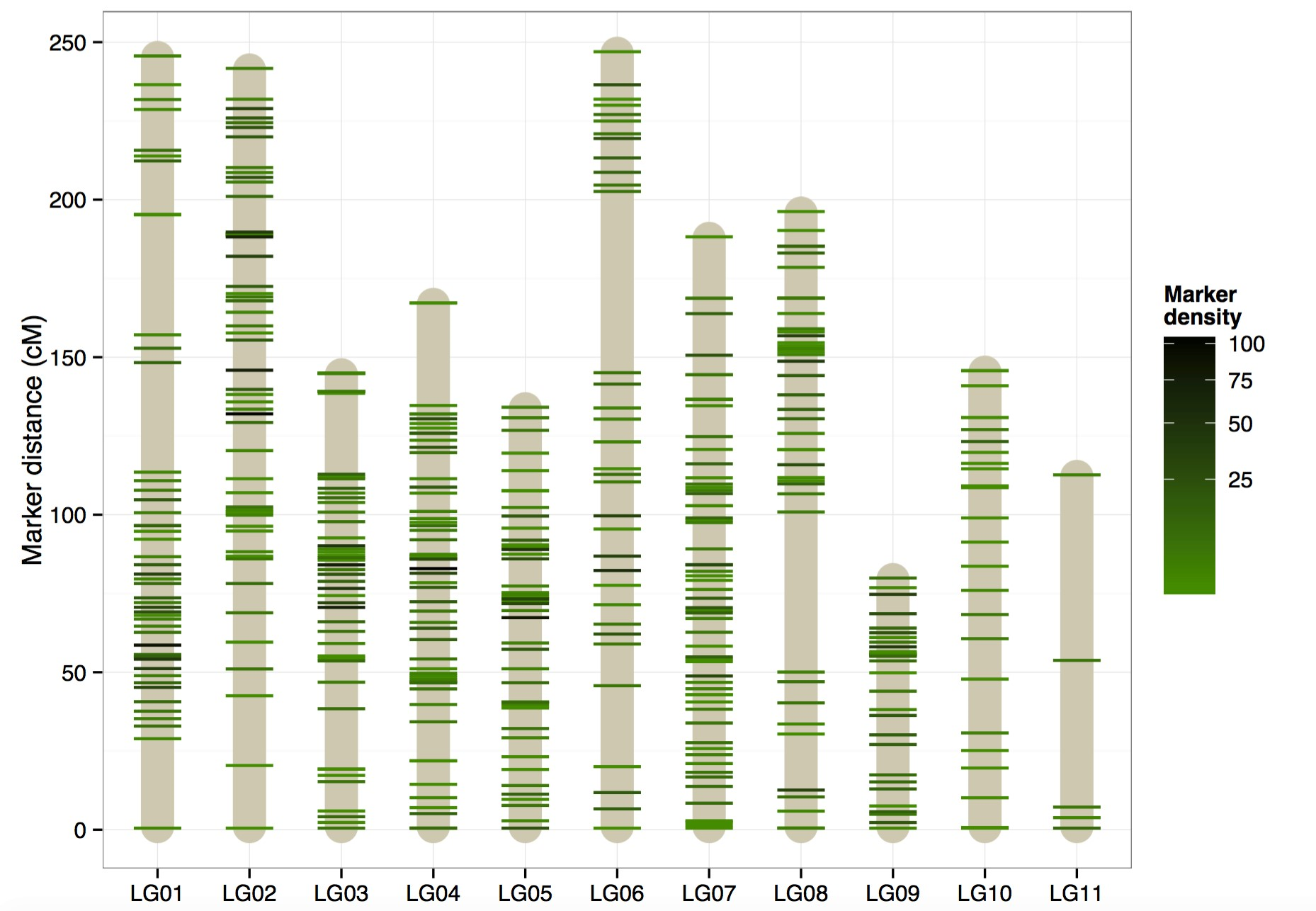

Supplement: S9 Fig — Marker density at each position is color coded with darker positions containing more markers. Markers on LG_10 are significantly sex-linked. (TIF) [file pgen.1007796.s017.tif]
